# Epigenetic mechanisms regulate sex differences in cardiac reparative functions of bone marrow progenitor cells

**DOI:** 10.1038/s41536-024-00362-2

**Published:** 2024-04-29

**Authors:** Charan Thej, Rajika Roy, Zhongjian Cheng, Venkata Naga Srikanth Garikipati, May M. Truongcao, Darukeshwara Joladarashi, Vandana Mallaredy, Maria Cimini, Carolina Gonzalez, Ajit Magadum, Jayashri Ghosh, Cindy Benedict, Walter J. Koch, Raj Kishore

**Affiliations:** 1 Aging and Cardiovascular Discovery Center, Lewis Katz School of Medicine, Temple University, Philadelphia, PA 19140 USA; 2 Department of Surgery, Division of Cardiovascular and Thoracic Surgery, Duke University School of Medicine, Durham, NC USA; 3 Fels Cancer Institute for Personalized Medicine, Lewis Katz School of Medicine, Temple University, Philadelphia, PA 19140 USA; 4 Department of Cardiovascular Sciences, Lewis Katz School of Medicine, Temple University, Philadelphia, PA 19140 USA

**Keywords:** Adult stem cells, Cell signalling

## Abstract

Historically, a lower incidence of cardiovascular diseases (CVD) and related deaths in women as compared with men of the same age has been attributed to female sex hormones, particularly estrogen and its receptors. Autologous bone marrow stem cell (BMSC) clinical trials for cardiac cell therapy overwhelmingly included male patients. However, meta-analysis data from these trials suggest a better functional outcome in postmenopausal women as compared with aged-matched men. Mechanisms governing sex-specific cardiac reparative activity in BMSCs, with and without the influence of sex hormones, remain unexplored. To discover these mechanisms, Male (M), female (F), and ovariectomized female (OVX) mice-derived EPCs were subjected to a series of molecular and epigenetic analyses followed by in vivo functional assessments of cardiac repair. F-EPCs and OVX EPCs show a lower inflammatory profile and promote enhanced cardiac reparative activity after intra-cardiac injections in a male mouse model of myocardial infarction (MI). Epigenetic sequencing revealed a marked difference in the occupancy of the gene repressive H3K9me3 mark, particularly at transcription start sites of key angiogenic and proinflammatory genes in M-EPCs compared with F-EPCs and OVX-EPCs. Our study unveiled that functional sex differences in EPCs are, in part, mediated by differential epigenetic regulation of the proinflammatory and anti-angiogenic gene CCL3, orchestrated by the control of H3K9me3 by histone methyltransferase, G9a/Ehmt2. Our research highlights the importance of considering the sex of donor cells for progenitor-based tissue repair.

## Introduction

Cardiovascular disease (CVD) is the leading cause of death in both men and women; however, the incidence of CVD in prepubescent, reproductive, and postmenopausal women is lower as compared with age-matched men^1,2^. Considering most diseases, global deaths were higher in men than women between 2007 and 2017. Amongst them, CVD-related morbidity and mortality in men were significantly higher than in women until the age of 80^1,3^. Based on evidence from some clinical trials, the incidence and deaths associated with CVD are markedly lower in women from the prepubescent stage up to three decades post-menopause as compared with aged-matched men. Sex hormones, specifically estrogen and its receptors have been suggested in several studies to be the primary cause of these cardioprotective differences in men and women^4–6^. Studies have shown that estrogen indirectly controls the vascular tone of the myocardial endothelium by nitric oxide (NO) regulation^7^. Notwithstanding, other factors such as adiponectin and silent mating-type information regulation 2 homolog 1 (SIRT1) can contribute to reactive oxygen species (ROS) reduction and NO sequestered endothelium-dependent relaxations^7,8^. Moreover, hormone therapy trials that were conducted using estrogen and progesterone have had mixed results^9–11^. A meta-analysis of 10 clinical trials, which included 38908 postmenopausal women, concluded that estrogen therapy had no effect on coronary events, myocardial infarction, revascularization, and total deaths^12^. Another review study that included an updated version of a Cochrane review published in 2013 aimed to assess the effects of hormone therapy for the prevention of cardiovascular disease in post-menopausal women^13^. The study included 19 randomized controlled trials with a total of 40,410 post-menopausal women, assessing outcomes such as all-cause mortality, cardiovascular death, myocardial infarction, stroke, venous thromboembolic events, and more. The key findings indicate that hormone therapy, whether used for primary or secondary prevention, did not confer protective effects against all-cause mortality, cardiovascular death, myocardial infarction, angina, or revascularization. Importantly, there was an increased risk of stroke and venous thromboembolic events in those receiving hormone therapy compared with those in the placebo or no treatment group^13^.

In myocardial injury, the recruitment of bone marrow cells into the ischemic myocardium in response to injury signals is well-known^14,15^. A well-studied example is that stromal-derived factor 1 alpha (SDF-1α) levels are upregulated in the heart tissue within an hour of ischemic injury, resulting in the migration of CXCR4^+^ bone marrow stem cells^16,17^. Another study showed that estrogen-regulated eNOS-mediated release of EPCs from the bone marrow, followed by matrix metalloprotease 9 (MMP-9), guided migration to the injured myocardium for vascular repair^18^. Several clinical trials have been conducted for CVDs using different stem cell populations from the bone marrow, such as EPCs, bone marrow mononuclear cells (BMMNCs), and mesenchymal stromal cells (MSC), with moderate outcomes^19–23^. Unfortunately, many of these clinical trials predominantly used male bone marrow stem cells. Based on the ACCRUE database (Meta-Analysis of Cell-based CaRdiac stUdiEs), the percentage of women in 13 different cell therapy studies for heart diseases ranged from 0 to 31%^24,25^. A subset analysis of cardiac function between women (*n* = 233) and men (*n* = 1019) who have undergone autologous stem cell therapy resulted in significant improvement in women^25^. Interestingly, the average age of women in these studies was higher than males (60 y for females vs. 56 y For males), suggesting that the therapeutic effects of cells were still evident during postmenopausal years in women^25^. While lack of information on the current hormonal status or possible hormone therapy in these women does not allow to claim sex hormone-independent mechanisms, nonetheless, data does suggest a better functional potency of female cells compared with age-match men. On the other hand, in preclinical studies, BM-derived mesenchymal stem cells (MSCs) from newborn female Sprague-Dawley rats had greater therapeutic efficacy than MSCs from newborn male rats in reducing neonatal hyperoxia-induced inflammation and vascular remodeling, suggesting that MSCs from females may be more potent at repair than MSCs from males^26^. At ages when there is greater homogeneity in sex hormone levels, some of these functional differences may be unaffected by female hormones. Only a limited number of studies have directly evaluated sex differences to show the functional superiority of female adult stem cells compared with male cells. A study showed that upon exposure to lipopolysaccharides (LPS), female mouse MSCs secreted low levels of tumor necrosis factor-alpha (TNFα) as compared with their male counterparts. These female MSCs also secreted higher amounts of vascular endothelial growth factor (VEGF) with LPS and hypoxia stimulation than male MSCs^27^. The same group later showed that intramyocardial injection of female mouse MSCs significantly improved left ventricular diastolic pressure (LVDP) and contractility compared with male mouse MSCs in a rat model of ischemic reperfusion (I/R) injury^28^. Another study showed that female rat MSCs mediated a significant increase in Bcl-xl/Bax ratio when injected intraperitoneally in a rat model of endotoxemia, leading to reduced myocardial apoptosis, inflammation, and dysfunction as compared with male MSCs^29^. The superiority of female stem/progenitor cells has primarily been associated with the influence of estrogen, estrogen receptors, and their downstream molecules rather than relying on mechanisms unaffected by sex hormones.

Although several studies show the positive effects of female sex hormones in enhancing bone marrow stem cell properties and reducing the risk of CVDs, this is an oversimplification. Women demonstrate greater cardioprotection than men even after a couple of decades post-menopause, suggesting possible cardioprotective mechanisms regardless of sex hormones. Sex differential epigenetic mechanisms, regardless of sex hormones, have been insufficiently studied as most studies focused on the role of estrogen, testosterone, and their receptors. Some of the fundamental processes of sex delineation, such as X chromosome inactivation, are regulated by epigenetic mechanisms^30^. Notably, euchromatic histone modifications such as H3K4me3, H3K27ac, and H3K36me3, as well as heterochromatic histone marks such as H3K9me3 and H3K27me3, were found to be significantly different between male and females, regulating X-chromosome inactivation as well as other functions^31^. Typically, the type of histone mark enriched on genomic regions regulates transcriptional gene repression or activation. The histone hallmarks of gene activation are euchromatin-associated, such as H3K16ac, H3K4me1/2/3, H3K9me1, etc. In contrast, heterochromatin-associated gene repressive marks are mainly H3K9me2/3, H3K27me3, and H3R2me, in addition to other posttranslational modifications such as biotinylation, SUMOylation, and citrullination^32^. Moreover, the role of histone modifiers such as histone methyltransferases, acetylases, and demethylases has been poorly understood in the context of cardiovascular disease. Our study aimed to investigate the cardioprotective epigenetic mechanisms of sex-specific EPCs following myocardial infarction (MI), regardless of sex hormone effects.

## Results

### Differential transcriptomics outlines sex differences in EPCs

**Fig. 1:**
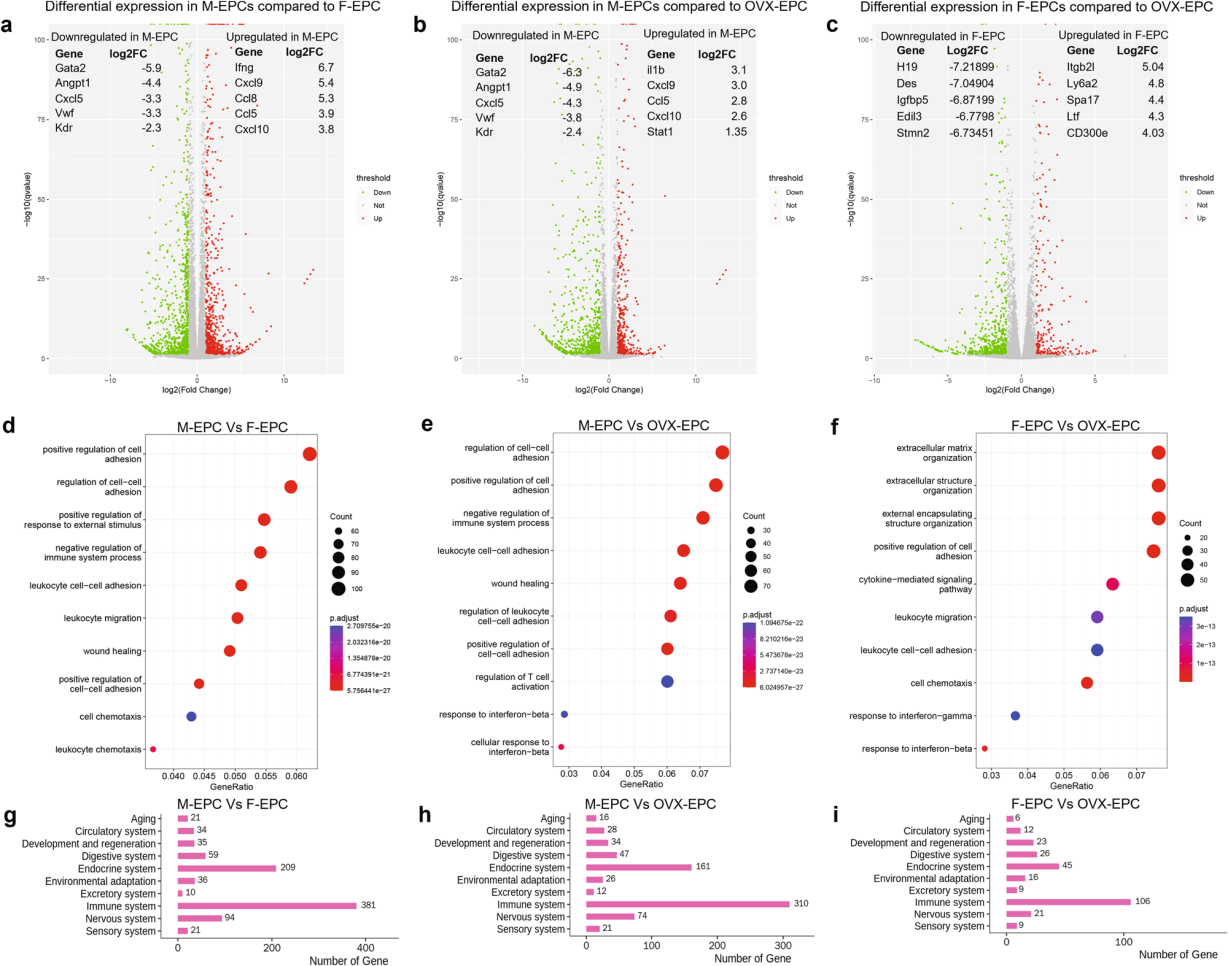
Analysis of whole-transcriptome RNA sequencing of male, female and OVX-EPCs. **a** Volcano plot of RNA sequencing analysis showing 2094 differentially expressed genes in M-EPC compared with F-EPC; Top 5 downregulated genes in M-EPCs: Gata2 (Log2FC: −5.9; *P* ≤ 1.31E−16), Angpt1 (Log2FC: −4.4; *P* ≤ 1.49E−24), CXCL5 (Log2FC: −3.3; *P* ≤ 7.59E−05), vWF (Log2FC: −3.3; *P* ≤ 0), and VEGFR2/Kdr (Log2FC: −3.3; *P* ≤ 9.94E-39); top 5 upregulated genes in M-EPCs: IFNγ (Log2FC: 6.7; *P* ≤ 1.14E−06), CXCL9 (Log2FC: 5.4; *P* ≤ 3.62E−22), CCL8 (Log2FC: 5.37; *P* ≤ 1.66E−83), CCL5 (Log2FC: 3.9; *P* ≤ 1.89E−211), and CXCL10 (Log2FC: 3.89; *P* ≤ 3.66E−100). **b** Volcano plot showing 1318 differentially expressed genes in M-EPCs compared with OVX-EPCs. Top 5 downregulated genes in M-EPCs: Gata2 (Log2FC: −6.3; *P* ≤ 2.61E−19), Angpt1 (Log2FC: −4.9; *P* ≤ 2.05E-30), CXCL5 (Log2FC: −4.3; *P* ≤ 0.0027), vWF (Log2FC: −3.8; *P* ≤ 0), and VEGFR2/Kdr (Log2FC: −2.4; *P* ≤ 2.00E-39); Top 5 upregulated genes in M-EPCs: IL1β (Log2FC: 3.1; *P* ≤ 3.86E-20), CXCL9 (Log2FC: 3.0; *P* ≤ 8.23E−76), CCL5 (Log2FC: 2.8; *P* ≤ 2.01E−163), Cxcl10 (Log2FC: 2.6; *P* ≤ 8.01E−52), Stat1 (Log2FC: 1.3; *P* ≤ 3.65E−138). **c** Volcano plot showing 812 differentially expressed genes in F-EPCs compared with OVX-EPCs. Top 5 downregulated genes in F-EPCs: H19 (Log2FC: −7.21; *P* ≤ 1.18E−07), Des (Log2FC: −7.04; *P* ≤ 1.46E−07), Igfbp5 (Log2FC: −6.8; *P* ≤ 4.93E−07), Edil3 (Log2FC: −6.7; *P* ≤ 6.99E−07) and Stmn2 (Log2FC: −6.7; *P* ≤ 9.49E−07). Top 5 upregulated genes in F-EPCs: Itgb2l (Log2FC: 5.04; *P* ≤ 0.0007), Ly6a2/Sca-1 (Log2FC: 4.8; *P* ≤ 0.001), Spa17 (Log2FC: 4.4; *P* ≤ 0.006), Ltf (Log2FC: 4.39; *P* ≤ 3.59E−20), Cd300e (Log2FC: 4.03; *P* ≤ 1.31E−05). **d** Gene ontology classification of M-EPC compared with F-EPC. **e** Gene ontology classification of M-EPC compared with OVX-EPC. **f** Gene ontology classification of F-EPC compared with OVX-EPC. KEGG pathway annotation; **g** M-EPC vs. F-EPC; **h** M-EPC vs. OVX EPC and **i** F-EPC vs. OVX-EPC.

The analysis of lineage-negative (Lin-) bone marrow cells revealed distinct populations, including Sca-1+, Sca-1−, CD31−, CD31+, and Sca-1+/CD31+ cells in male (Supplementary Fig. 1a), female (Supplementary Fig. 1d), and OVX mice (Supplementary Fig. 1g). We expanded magnetically selected Sca-1+/CD31+ cells in culture, confirming their EPC phenotype through morphology (Supplementary Figure 1b, e, h) and AcLDL-DiI uptake (Supplementary Fig. 1c, f, I; Supplementary Fig. 11). Whole-genome RNA transcriptomic analysis revealed 2094 differentially expressed genes between M-EPCs and F-EPCs (Fig. 1a; Supplementary Fig. 2a) and 1318 differentially expressed genes between M-EPCs and OVX-EPCs (Fig. 1b; Supplementary Fig. 2b). In contrast, only 812 genes showed differential expression between F-EPCs and OVX-EPCs despite variable estrogen status (Fig. 1c; Supplementary Fig. 2c). Notably, proinflammatory chemokines and cytokines like IFNγ, CXCL9, CCL8, CCL5, CXCL10, etc. were significantly upregulated in M-EPCs as compared with F-EPCs. (Fig. 1a). The top downregulated genes in M-EPCs as compared with F-EPCs were endothelial-specific, proangiogenic genes such as Gata2, Angiopoietin 1 (Angpt1), CXCL5, Von-Willebrand factor (Vwf) and Vascular endothelial growth factor receptor 2 (VEGFR2/Kdr) (Fig. 1a). The top 5 upregulated genes in M-EPCs compared with OVX-EPCs were Interleukin 1 beta (IL1β), CXCL9, CCL5, CXCL10 and Stat1 (Fig. 1b). Significantly downregulated genes in M-EPCs compared with OVX-EPCs were identical to the comparison with F-EPCs (Fig. 1b). Minimal differential expression was observed between F-EPCs and OVX-EPCs, indicating that the absence of female sex hormones had a minor impact on EPC transcriptomic profiles (gray area) (Fig. 1c). The top 5 significantly downregulated genes in F-EPCs compared with OVX-EPCs were H19, Des, Igfbp5, Edil3, and Stmn2 (Fig. 1c). The top 5 differentially upregulated genes in F-EPCs compared with OVX-EPCs were Itgb2l, Ly6a2, Spa17, Ltf and CD300e (Fig. 1c). Functional enrichment analysis supported these findings, highlighting genes associated with leukocyte cell-cell adhesion, leukocyte migration, and leukocyte chemotaxis in M-EPCs compared with F-EPCs (Fig. 1d) and OVX-EPCs (Fig. 1e), indicating the proinflammatory phenotype of M-EPCs. Additionally, the number of genes associated with leukocyte migration, cell-cell adhesion, and cell chemotaxis was significantly lower between F-EPCs and OVX-EPCs (Fig. 1f). Further analysis using KEGG pathway annotation showed that 381 immune system-related genes were upregulated in M-EPCs compared with F-EPCs (Fig. 1g), and 310 immune system genes were upregulated in M-EPCs compared with OVX-EPCs (Fig. 1h). In contrast, only 106 immune system genes were differentially upregulated in F-EPCs compared with OVX-EPCs (Fig. 1i). Additionally, analysis of the different EPC sex dimorphic groups using MA-plot revealed that M-EPCs exhibited high expression of C3 compared with F-EPCs and OVX-EPCs (Supplementary Figure 2d-e). Conversely, CD74, a type 2 transmembrane protein, was downregulated in F-EPCs compared with OVX-EPCs (Supplementary Fig. 2f). Comparison of sex differential EPCs using top gene ontology (Top GO) analysis of biological process (BP) showed that the top genes in M-EPCs were significantly associated with leukocyte migration, cell migration and leukocyte chemotaxis, leukocyte cell-cell interactions, T-cell regulation, etc. compared with F-EPCs (Supplementary Fig. 3a) and OVX-EPCs (Supplementary Fig. 3b). In contrast, Top GO in F-EPCs were associated with extracellular structure, external encapsulation and extracellular matrix interaction compared with OVX-EPCs (Supplementary Fig. 3c). Evaluation of the top 100 significantly differential genes using STRING highlighted strong interactions among proinflammatory genes, such as IFNγ, CCL5, CCL8, CXCL9, and CXCL10 in M-EPCs compared with F-EPCs (Supplementary Fig. 2g). Similarly, strong STRING interactions were found between CXCL9, CXCL10, CCL5, IL1β, and NOS2 in M-EPCs compared with OVX-EPCs (Supplementary Fig. 2h). In contrast, substantially fewer interactions were found in F-EPCs, with the primary interaction being between CCL2 and CCL7 with their receptors (Supplementary Fig. 2i).

### Female EPCs exhibit a reparative secretome and promote angiogenesis in vitro independent of estrogen effects

**Fig. 2:**
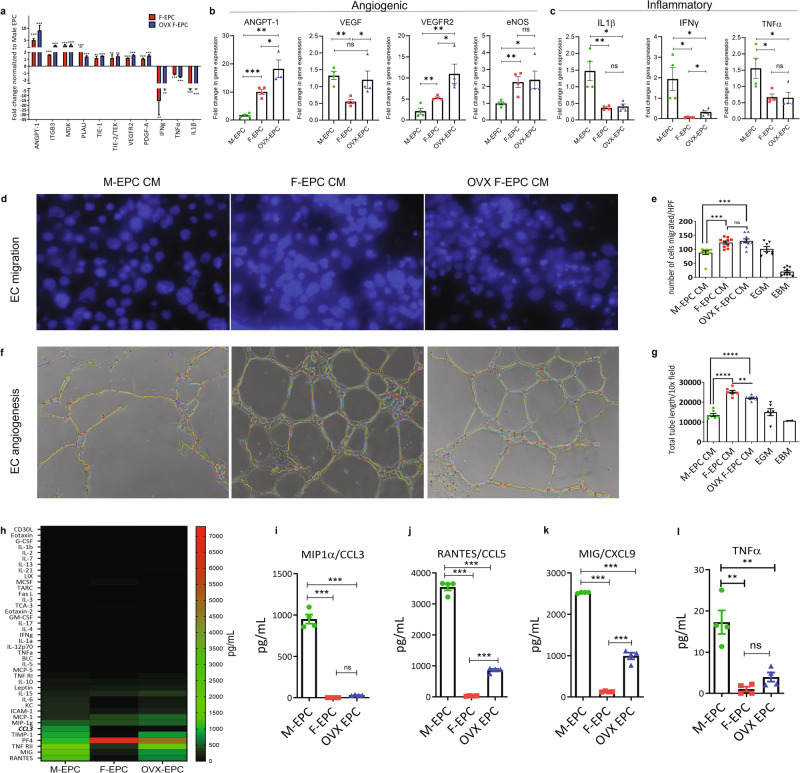
Angiogenic and inflammatory activity of sex differential EPCs. **a** Proangiogenic and inflammatory gene expression array of F-EPC and OVX EPCs normalized to the expression of M-EPC. **b** qPCR gene expression of key angiogenic factors, Angpt-1, VEGF, VEGFR2/Kdr, and eNOS. **c** qPCR gene expression of inflammatory factors, IL1β, IFNγ, and TNFα. F-EPC and OVX-EPC CM promoted significantly higher migration (**d**, **e**) and tube formation (**f**, **g**) of MCECs compared with M-EPCs. Lesser differences in function were observed between F-EPC and OVX-EPC (**d**–**g**). EGM and EBM (basal medium) were used as positive and negative controls, respectively. **h** Heat map representation of the protein dot blot shows high expression of inflammatory factors in M-EPC CM compared with F-EPC and OVX EPC CM. Graphical representation of Inflammatory factors based on quantitative ELISA array shows M-EPCs secrete high levels of CCL3 (**i**), CCL5 (**j**), CXCL9 (**k**), and TNFα (**l**). The total magnification of 2000× and 1000× was used for EC migration and tube formation, respectively. *P < 0.01; **P < 0.001; ***P < 0.0005; ****P < 0.0001. Data are shown as mean ± s.e.m.

Evaluation of proangiogenic and inflammatory genes using a gene array in sex differential EPCs show high levels of proangiogenic genes such as Angpt-1, Integrin subunit β3 (ITGB3), Midkine (MDK), Platelet-derived growth factor A (PDGF-A), and VEGFR2, etc., and low expression of proinflammatory interferon-gamma (IFNγ), tumor necrosis factor-alpha (TNFα) and interleukin-1-beta (IL1β) in F-EPCs and OVX-EPCs compared with M-EPCs (Fig. 2a). Using qRT-PCR, we further confirmed the gene expression of key angiogenic genes reported extensively in literature. We found significantly high expression of Angpt-1, VEGFR2/Kdr and eNOS in F-EPCs and OVX-EPCs compared with M-EPCs (Fig. 2b). Exceptionally, gene expression of VEGF in F-EPCs was lower compared with M-EPCs and OVX-EPCs (Fig. 2b). We then confirmed that gene expression levels of IL1β, IFNγ and TNFα were significantly higher in M-EPCs compared with F-EPCs and OVX-EPCs (Fig. 2c). Importantly, no differences in the expression of the three inflammatory genes were found between F-EPCs and OVX-EPCs (Fig. 2c).

Next, we compared the in vitro angiogenic functions elicited by sex-dimorphic EPCs. The conditioned medium (CM) was collected from Sca-1^+^/CD31^+^ selected sex differential EPCs and was used to assess the migration and angiogenic tube formation activity using MCECs. F-EPC and OVX EPC CM promoted significantly higher EC migration compared with M-EPC CM (Fig. 2d, e). There was no significant difference between the F-EPC and OVX EPC secretome in promoting endothelial cell migration. The F-EPC and OVX EPC CM induced robust tube formation with significantly longer tube lengths (Fig. 2f, g) compared with M-EPC CM. Both F-EPCs and OVX-EPCs CM elicited efficient angiogenic functions, indicating no dependency on estrogen mechanisms.

The high expression of proinflammatory factors IL1β, IFNγ, and TNFα in M-EPCs and their low expression in both F-EPCs and OVX-EPCs prompted us to evaluate their inflammasome further. We quantified a comprehensive panel of 40 proinflammatory factors in the secretome of EPCs. We found that, overall, M-EPCs secreted higher levels of proinflammatory factors such as RANTES/CCL5, MIG/CXCL9, TNFRII, TIMP-1, CCL3, ICAM-1, KC and TNFα compared with F-EPCs and OVX-EPCs (Fig. 2h). Amongst the proinflammatory factors, CCL3 was highly secreted by the male EPCs in large quantities (951.7 ± 109.9 pg/mL) compared with significantly low secretion in F-EPCs and OVX EPCs (30.2 ± 23 pg/mL; *P* ≤ 0.0001) (Fig. 2i). Some of the other pernicious inflammatory factors, such as CCL5 (Fig. 2j), CXCL9 (Fig. 2k), and TNFα (Fig. 2l), were secreted in significantly high levels by the M-EPCs compared with F-EPCs and OVX-EPCs. Upon examining the magnitude of angiogenic and proinflammatory factors, fewer differences were observed between F-EPCs and OVX-EPCs, highlighting sex hormone-independent regulation of their paracrine secretome.

### Female EPCs elicit superior post-MI reparative properties independent of estrogen influence

**Fig. 3:**
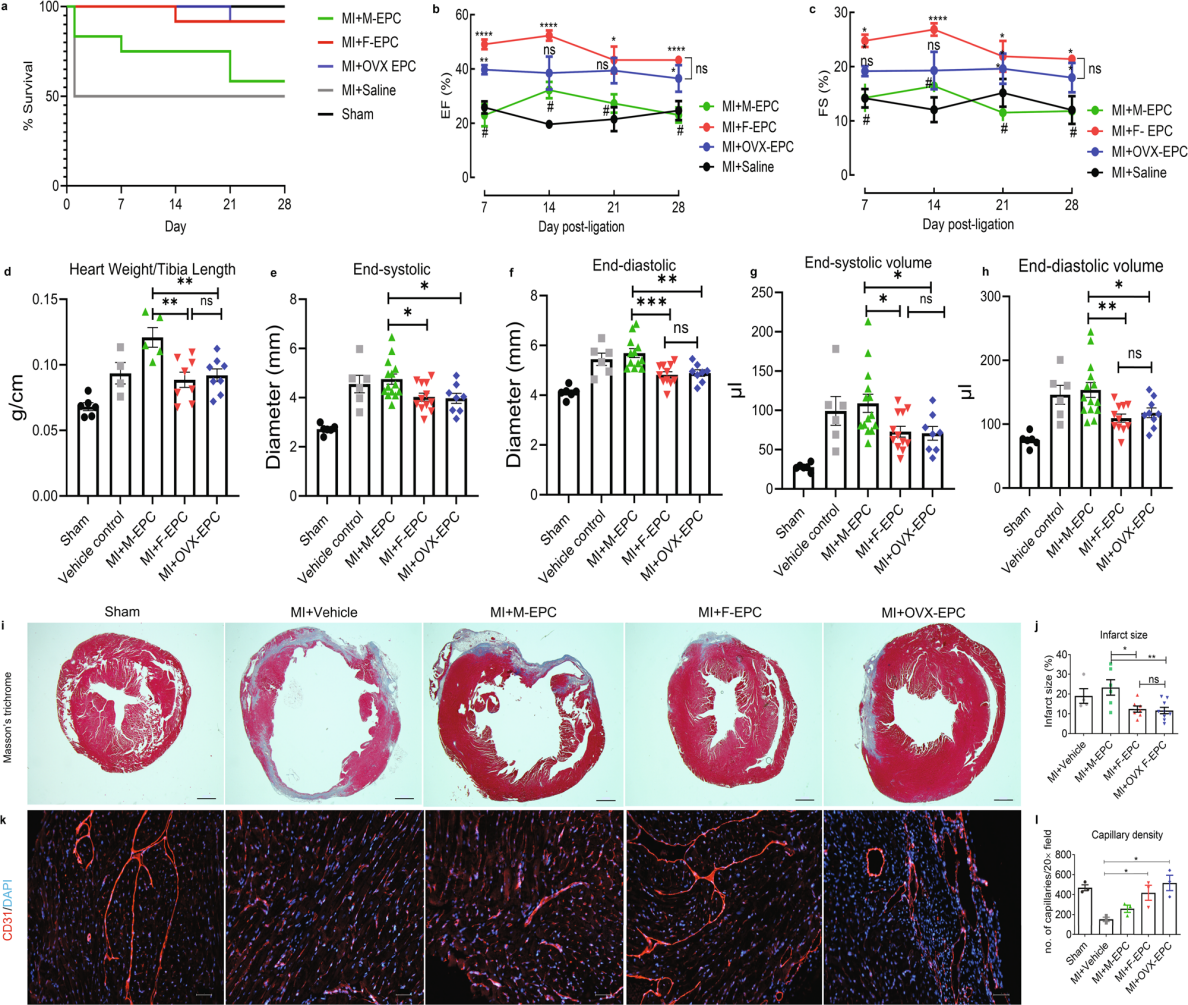
Post-MI reparative properties of male, female, and OVX-EPCs. **a** Transplantation of F-EPC and OVX-EPCs resulted in 90% survival of mice compared with 60% survival in recipients of M-EPCs. **b** F-EPC injection resulted in significantly improved %EF evaluated on all days, 7 (****P < 0.0001 vs. M-EPC), 14 (****P < 0.0001 vs. M-EPC), 21 (*P < 0.01 vs. M-EPC) and 28 (****P < 0.0001 vs. M-EPC). OVX-EPC injection significantly improved %EF on days 7 (**P < 0.001 vs. M-EPC) and 28 (*P < 0.01 vs. M-EPC) post-MI. **c** F-EPC injection significantly improved %FS on days, 7 (*P < 0.01 vs. M-EPC), 14 (****P < 0.0001 vs. M-EPC), 21 (*P < 0.01 vs. M-EPC) and 28 (*P < 0.01 vs. M-EPC). OVX-EPC injection significantly improved %FS on days 21 (*P < 0.01 vs. M-EPC) and 28 (*P < 0.01 vs. M-EPC). F-EPC and OVX-EPC injection in post-MI mice maintained significantly lower HW/TL (**d**), end-systolic diameter (**e**), end-diastolic diameter (**f**), end-systolic volume (**g**) and end-diastolic volume (**h**) compared with M-EPCs. No significant differences were observed between F-EPCs and OVX-EPCs in all the measured cardiac functions (**a**–**f**). **i**, **j** Masson’s trichrome staining showed significantly higher infarct size in the MI + M-EPC injected group compared with the MI + F-EPC and MI + OVX EPC injected group; scale bar = 1000 μm. **k**, **l** Angiogenic evaluation by measurement of capillary density using immunofluorescent CD31 staining shows a significantly higher number of capillaries in F-EPC and OVX-EPC injected groups compared with M-EPCs and Vehicle control group. Data are shown as mean ± s.e.m. Cohort; Sham (*n* = 6), Vehicle (*n* = 8), M-EPC (*n* = 12), F-EPC (*n* = 12), OVX-EPC (*n* = 12). *P < 0.01; **P < 0.001; ***P < 0.0005; ****P < 0.0001; scale bar = 50 μm. Data are shown as mean ± s.e.m.

Prior to comparing the therapeutic efficacy of Sca-1+/CD31+ EPCs, we wanted to evaluate if female endogenous bone marrow cells migrate to the ischemic myocardium post-MI to elicit enhanced remodeling compared with male bone marrow irrespective of female sex hormone influence. Therefore, we conducted cross-sex bone marrow transplantation (BMT) experiments. Bone marrow from male, female, and OVX C57BL/6-Tg(UBC-GFP)30Scha/J mice were transplanted into irradiated C57BL/6 J male mice, and the graft was allowed five weeks for reconstitution. The recipient male mice then underwent MI surgery to evaluate the dynamics of sex-dimorphic bone marrow mobilization and repair in the ischemic myocardium. We observed high mobilization of GFP^+^ cells in post-MI hearts of all male mice reconstituted with varied bone marrow (Supplementary Fig. 4a–c). Although not significant, we observed a trend of improved cardiac function in post-MI mice transplanted with female BM compared with mice with male BM (Supplementary Fig. 4). Strikingly, male recipients of OVX BM showed significantly improved %EF and %FS compared with male (**P = 0.002) and female (*P = 0.02) BM chimeric mice. Mice transplanted with female and OVX female BM showed smaller infarct size and significantly protected capillaries compared with mice with male BM (Supplementary Fig. 4). Previous studies have shown that 20% of the cells that migrate to the injured myocardium are Sca-1^+^/CD31^+^/VEGFR2^+^ EPCs that attempt to repair ischemic tissue^33^. These results encouraged us to further evaluate the differential cardiac reparative role of sex dimorphic EPCs in post-MI repair regardless of female sex hormone influence. Culture-expanded male, female, and OVX Sca-1/CD31 EPCs (2 × 10^5^ cells/mouse) were intramyocardially transplanted into male hearts post LAD ligation to compare their cardiac reparative functions post-MI (Fig. 3). Post-MI therapeutic outcomes, irrespective of sex hormone status, were significantly improved with the intracardiac transplantation of F-EPCs compared with their male counterparts. Transplantation of F-EPCs and OVX-EPCs resulted in 90% survival of mice compared with 60% survival in the M-EPCs injected group (Fig. 3a). We found that cardiac functions were significantly preserved with the injection of F-EPCs and OVX EPCs in post-MI male mice. F-EPC injection significantly preserved %EF (Fig. 3b) and %FS (Fig. 3c) compared with M-EPC on days 7, 14, 21 and 28 of echocardiographic assessment. OVX-EPC injection also showed significant preservation of %EF (Fig. 3b) on days 7 and 28 and %FS (Fig. 3c) on days 21 and 28 compared with M-EPC injected mice. Importantly, no significant differences between the F-EPC and OVX EPC treatment groups suggested no influence of sex hormones. Post-MI male mice injected with F-EPCs and OVX-EPCs exhibited significantly lower heart weight/tibia length (HW/TL) compared with M-EPC-injected mice (Fig. 3d). The HW/TL was considerably higher in M-EPC-injected post-MI mice compared with the vehicle-injected group (Fig. 3d). Corresponding to the heart weight, LV end-systolic (LVESD) (Fig. 3e) and end-diastolic diameter (LVEDD) (Fig. 3f) as well as LV end-systolic (LVESV) (Fig. 3g) and end-diastolic volumes (LVEDV) (Fig. 3h) were found to be significantly higher in M-EPC injected group compared with F-EPC and OVX-EPC group of mice.

Histological analysis using Masson’s trichrome staining showed reduced fibrosis and infarct size (Fig. 3i, j) in post-MI hearts injected with female and OVX EPCs, suggesting greater cardiac contractility compared with the male EPC injected group and the vehicle group. Notably, the left ventricular wall was well-protected from further ischemic damage in the F-EPC and OVX-EPC injected group compared with the M-EPC group (Fig. 3i). CD31 staining of vasculature in the post-MI heart tissues injected with female and OVX female EPCs showed significantly increased angiogenesis, evidenced by increased capillary density in the ischemic border zone compared with mice treated with male EPCs or vehicle (Fig. 3k, l) (Supplementary Fig. 4a).

### M-EPC injection post-MI promotes lymphocyte infiltration and inflammation

**Fig. 4:**
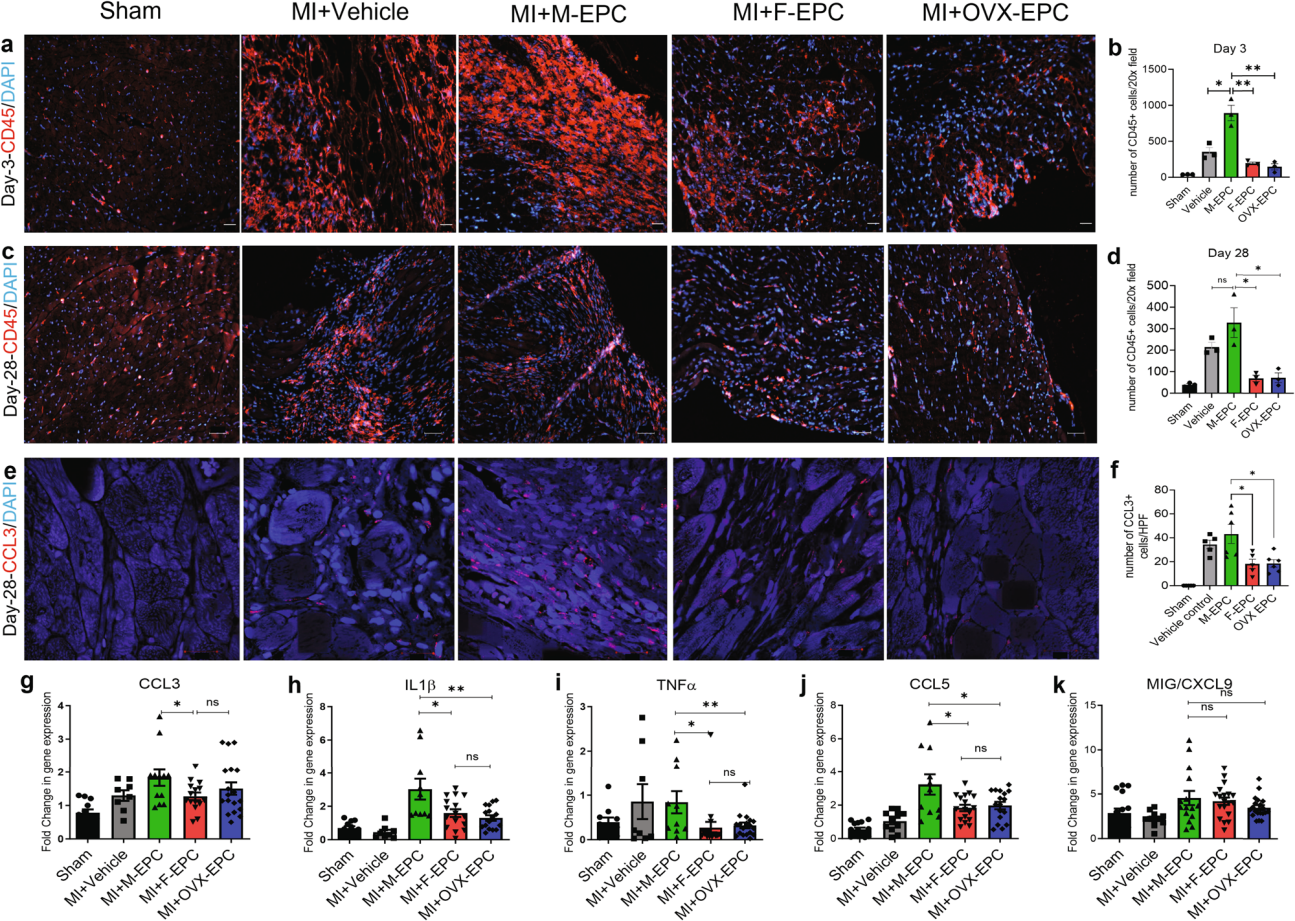
Leukocyte infiltration and tissue inflammation. **a**, **b** Injection of M-EPC prompted high infiltration of CD45+ leukocytes compared with vehicle control and female injection groups on day 3 and day 28 post-MI. F-EPC and OVX EPC resulted in significantly low CD45+ cell infiltration on day 3 (**P = 0.003) and day 28 post-MI (**P = 0.003) (**c**, **d**); scale bar = 50 μm. **e**, **f** On day 28 post-MI, we found that injection of M-EPCs resulted in high localization of CCL3 compared with low levels of CCL3 in heart tissues of other groups; scale bar = 20 μm. M-EPC injection post-MI resulted in significantly high mRNA expression of proinflammatory genes: CCL3 (**g**), IL1β (**h**), TNFα (**i**), and CCL5 (**j**) compared with F-EPCs and OVX-EPCs. Levels of CXCL9 (**k**) remained unchanged in the cell treatment groups. *P < 0.01; **P < 0.001. Data are shown as mean ± s.e.m.

Post-MI, immune cells of hematopoietic origin infiltrate the injured myocardium within 3–4 days in mice^34^. Therefore, we investigated the effect of transplanting M-EPC, F-EPC, and OVX-EPC intramyocardial transplantation post-MI on leukocyte infiltration and tissue inflammation (Fig. 4). Immunohistochemical evaluation showed that intra-cardiac Injection of M-EPC resulted in the significantly greater infiltration of CD45+ cells predominantly localized in the ischemic border zone compared with F-EPC and OVX EPC on day 3 post-MI (Fig. 4a, b; Supplementary Fig. 5b). The extent of lymphocyte infiltration in the M-EPC-injected mice was significantly higher than that of mice that received vehicle injections, indicating the role of injected EPCs in orchestrating the infiltration of CD45+ cells. On day 28, the number of CD45+ lymphocytes remained significantly high in the cardiac tissues of mice injected with M-EPC compared with post-MI heart tissues injected with F-EPC and OVX-EPCs (Fig. 4c, d; Supplementary Fig. 5c). Since M-EPCs secrete high levels of CCL3 compared with significantly low levels by F-EPCs and OVX-EPCs, we evaluated the expression of CCL3 in post-MI hearts of the different treatment groups. We found high levels of CCL3 expression in the post-MI heart tissues of mice injected with M-EPCs compared with lower expression in mice hearts injected with F-EPC, OVX-EPC, or vehicle (Fig. 4d, f).

To further evaluate the inflammatory milieu post-transplantation in response to sex-specific EPC treatments, we examined cardiac tissue expression of proinflammatory cytokines on day 28. Post-MI hearts injected with M-EPCs showed significantly augmented expression of proinflammatory genes, CCL3 (Fig. 4g), IL-1β (Fig. 4h), TNFα (Fig. 4i), CCL5 (Fig. 4j) and CXCL9 (Fig. 4k) compared with F-EPC and OVX-EPC. Overall, we observed that the expression of most proinflammatory genes was higher in hearts injected with M-EPCs (Fig. 4g). We speculated that the presence of injected EPCs may be contributing to the differential inflammatory milieu. Therefore, we evaluated and confirmed the presence of GFP^+^ EPCs on day 28 post-MI tissues, likely influencing sex differential inflammation dynamics (Supplementary Fig. 5d). We also assessed the presence of proinflammatory molecules in the serum of mice from the various treatment groups on Day 3 (Supplementary Fig. 5e) and Day 28 (Supplementary Fig. 5f) post-MI. Overall, we found that the serum of mice injected with M-EPCs had higher levels of proinflammatory factors, especially IFNγ and IL-6, on day 3 (Supplementary Fig. 5e). Of notable interest, on day 28, the serum of mice injected with M-EPCs had substantially low levels of IL-10, an anti-inflammatory molecule known to be secreted by M2 macrophages, suggesting that polarization of macrophages to the M2 phenotype was limited in the M-EPC injected group (Supplementary Fig. 5f).

### EPCs demonstrate sex differences in regulating monocyte migration and polarization in vitro and in vivo

**Fig. 5:**
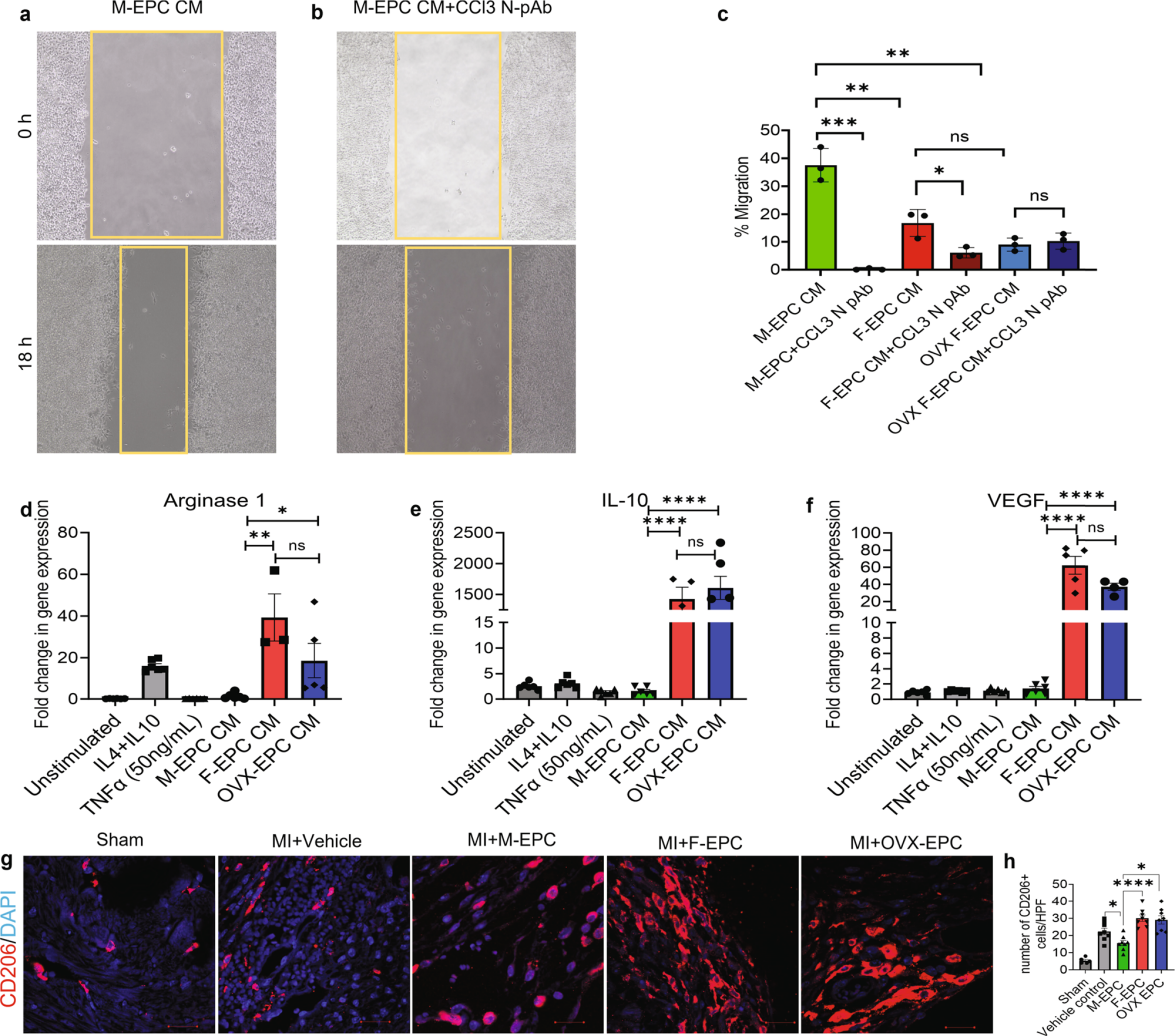
Monocyte migration and polarization. **a** Male EPC CM containing high levels of inflammatory cytokines promoted greater migration of monocytes at 18 h of inducing them in a scratch assay in vitro. **b**, **c** Inhibition of CCL3 using CCL3-neutralizing polyclonal antibody (CCL3-N-pAb) in M-EPC CM significantly inhibited the migration of monocytes. **c** Lower migration of monocytes was promoted by F-EPC and OVX-EPC. Inhibition of CCL3 in F-EPC and OVX EPC had low or no effect on monocyte migration. F-EPC and OVX-EPC CM promoted polarization of monocytes into a macrophage M2-like phenotype, resulting in upregulated expression of Arginase 1 (**d**), IL-10 (**e**), and VEGF (**f**). Male EPC did not promote alternative switching of monocytes to the M2 phenotype. **d**–**f** TNFα was used on activated monocytes to induce the M1 phenotype switch as a positive control. IL-4 and IL-10 stimulated monocytes were used as a positive control for the M2 phenotype. **g**, **h** significantly high numbers of CD206^high^M2 cells were found in heart tissue sections of mice injected with F-EPC and OVX-EPC compared with the M-EPC injected group. Vehicle and sham hearts were also stained with CD206 as controls. *P < 0.01; **P < 0.001; ***P < 0.0005; ****P < 0.0001; scale bar = 20 μm. Data are shown as mean ± s.e.m. *n* = 3–7.

Our previous analysis found that proinflammatory cytokines and chemokines such as CCL3 were highly synthesized and secreted by the M-EPCs compared with very low secretion by F-EPCs and OVX EPCs (Fig. 1). Therefore, we hypothesized that high levels of proinflammatory factors secreted by the M-EPCs, especially CCL3, largely contribute to leukocyte infiltration post-MI. To evaluate the same function in vitro, we used CM from male, female, and OVX EPCs to assess in vitro monocyte migration (Fig. 5a, b). We observed that M-EPC CM significantly promoted the migration of monocytes compared with F-EPC and OVX EPC CM (Fig. 5a, b). Furthermore, neutralization of CCL3 in the M-EPC CM using CCL3-N-pAb significantly abrogated the in vitro migration of monocytes (Fig. 5a, b), confirming that CCL3 is a significant contributing factor to monocyte migration. Importantly, no differences in promoting monocyte migration were observed between the F-EPC and OVX-EPC secretomes (Fig. 5a, b).

Next, we checked if the differential EPC secretomes could regulate monocyte polarization and elicit a secondary response to EPC repair mechanisms (Fig. 5d–f). We found that F-EPC and OVX-EPC promoted the polarization of activated monocytes to a macrophage M2-like phenotype by upregulating the expression of arginase-1 (Fig. 5d), IL-10 (Fig. 5e) and VEGF (Fig. 5f), all of which are known to be expressed in high levels by M2 macrophages. On the contrary, we found that CM from M-EPCs did not promote polarization toward M2-like macrophages (Fig. 5c–f). Consequently, we examined the number of CD206^hi^ M2-like cells in the cardiac tissues of mice injected with different EPCs. Consistent with our in vitro findings, we found significantly higher numbers of CD206^hi^ cells in post-MI hearts of mice injected with F-EPCs and OVX-EPCs compared with low numbers in the M-EPCs-injected group, suggesting that M-EPC injection may inhibit macrophage phenotype-switching and inherent repair mechanisms (Fig. 5g, h). On the contrary, F-EPCs, irrespective of estrogen dependence, are likely to induce alternative switching of macrophages to further promote repair in the ischemic myocardium. These data support our finding of lower IL-10 levels in the serum of mice injected with M-EPCs at day 28 (Supplementary Fig. 5f).

### Differential genomic H3K9me3 occupancy influences sex differences in EPCs

**Fig. 6:**
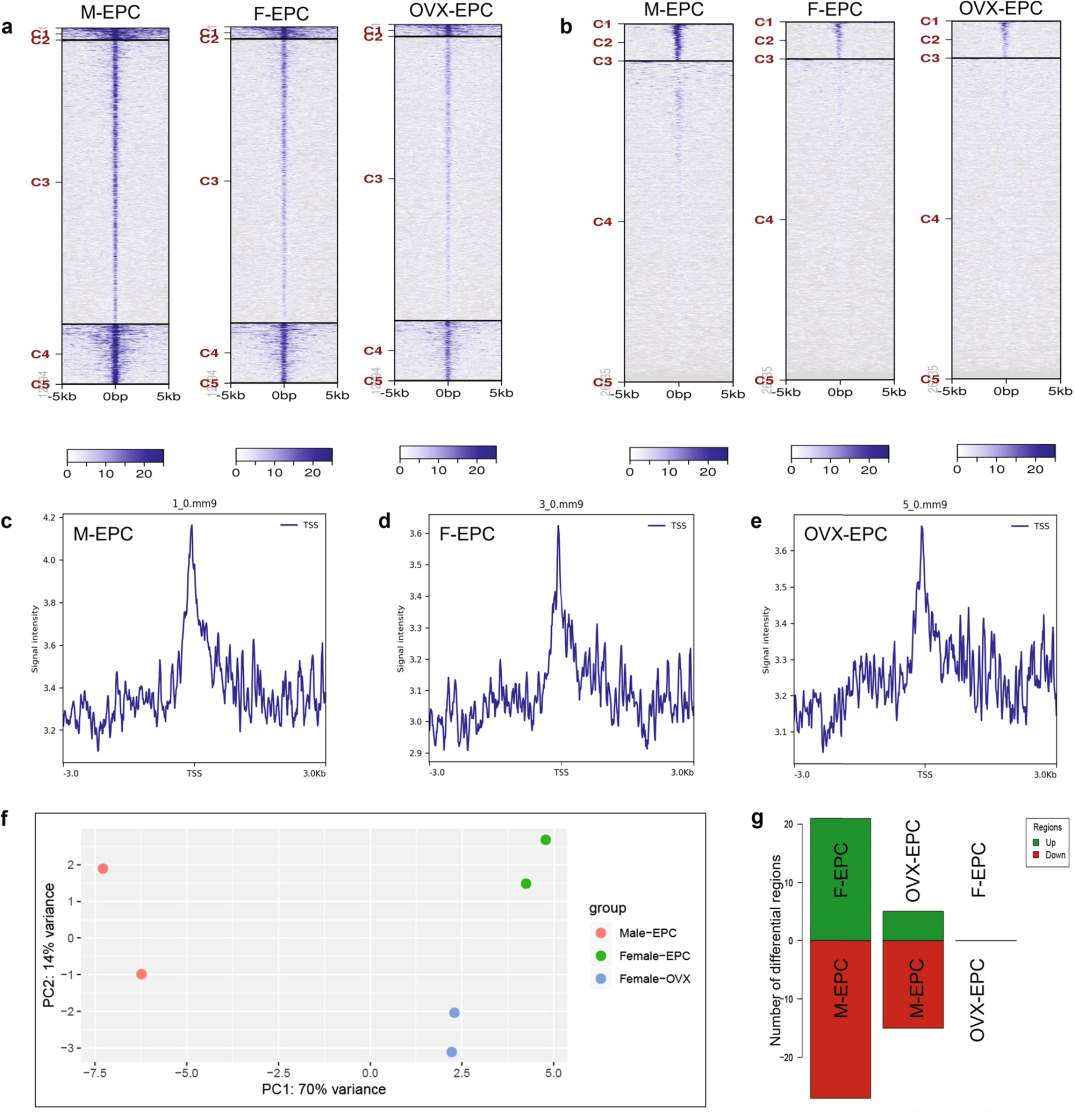
Whole genome CUT&Tag sequencing of M-EPC, F-EPC, and OVX EPC. Heat map showing whole-genome H3K9me3 occupancy in merged regions (**a**) and TSS/Promoter regions (**b**) in male, female, and OVX EPCs within a −5 kb to 5 kb window. Peaks representing signal frequency at TSS of M-EPC (**c**), F-EPC (**d**), and OVX-EPC (**e**). Principal component analysis (PCA) showing substantially high variance in H3K9me3 genomic occupancy between male EPCs and female EPCs, irrespective of female sex hormones (**f**). Significantly less variance is seen between F-EPCs and OVX-EPCs. Bar graph indicating a high number of differentially methylated regions between M-EPCs and F-EPCs or OVX-EPCs (**g**). Least differentially methylated regions were found between F-EPCs and OVX-EPCs.

Histone modifications, particularly H3 modifications, have been well-studied and implicated in gene silencing or activation. We postulated that differential angiogenic and inflammatory functions of male and female EPCs are not due to female sex hormone influence but due to underlying epigenetic modifications. Initially, we estimated posttranslational modifications of H3 using a quantitative multiplex ELISA array approach using histone extracts of M-EPCs, F-EPCs, and OVX-EPCs (Supplementary Fig. 6a). We found that the expression of several H3 marks was significantly higher in M-EPCs than in F-EPC and OVX-EPCs. Although we initially found sex differential expression of several histone modifications, we chose to focus on the genomic distribution of H3K9me3 further, as it is a constitutive gene repression mark and differentially expressed in male, female, and OVX EPCs (Supplementary Fig. 6b). We then subjected the three different EPCs to whole-genome CUT&Tag-IT^TM^ sequencing, specific to H3K9me3. We found that M-EPCs had marked genome-wide enrichment of H3K9me3 specific regions compared with F-EPCs and OVX EPCs based on the IGV browser screenshot (Supplementary Fig. 6c) as well as the genome-wide heat map (Fig. 6a). Upon further analysis of only the promoter/TSS regions between the EPC groups, we found significantly high H3K9me3 occupancy at TSS/promoter regions in M-EPCs compared with F-EPCs and OVX-EPCs (Fig. 6b), supported by higher signal intensity of H3K9me3 peaks in M-EPCs (Fig. 6c) at the TSS between −3kb and 3 kb compared with F-EPCs (Fig. 6d) and OVX-EPCs (Fig. 6e). Pie-chart analysis of the H3K9me3 occupancy on different locations on the genome showed that M-EPC promoter regions were highly methylated (31.88%) (Supplementary Fig. 7a) compared with F-EPC (19.99%) (Supplementary Fig. 7b) and OVX EPC (22.02%) (Supplementary Fig. 7c). Overall, less variation was observed between the F-EPC and OVX EPC despite the presence or absence of sex hormones. Gene ontology (GO) analysis showed that M-EPC were highly methylated in most biological processes, molecular functions, and cellular components, indicating that M-EPC may be less functional than F-EPC and OVX EPC (Supplementary Fig. 7d, e). For instance, H3K9me3 peak annotations associated with cytoplasm, nucleus, nucleolus and protein binding were substantially higher in M-EPCs (Supplementary Fig. 7d) compared with F-EPCs (Supplementary Fig. 7e) and OVX-EPCs (Supplementary Fig. 7f). KEGG-pathway analysis showed a differential methylation pattern of pathways between male and female EPCs (Supplementary Fig. 7g–i). M-EPCs showed lesser H3K9me3 occupation in TNF pathway genes with a gene ratio ≤0.025 (Supplementary Fig. 7g). In contrast, high H3K9me3 occupancy was seen in TNF pathway genes with a gene ratio of ~0.03 (Supplementary Fig. 7h). Moreover, in the F-EPCs, MAPK signaling genes were enriched by H3K9me3 occupancy with a gene ratio of ~0.06 (Supplementary Fig. 7h). In the OVX-EPCs, H3K9me3 enrichment of TNF signaling pathway genes was higher than M-EPCs (gene ratio ≥;=0.025) (Supplementary Fig. 7i). In addition, T cell receptor pathway genes were enriched with H3K9me3 with a gene ratio of 0.03 (Supplementary Fig. 7i).

### G9a/Ehmt2 histone methyltransferase controls the expression and secretion of inflammatory and reparative factors in EPCs

**Fig. 7:**
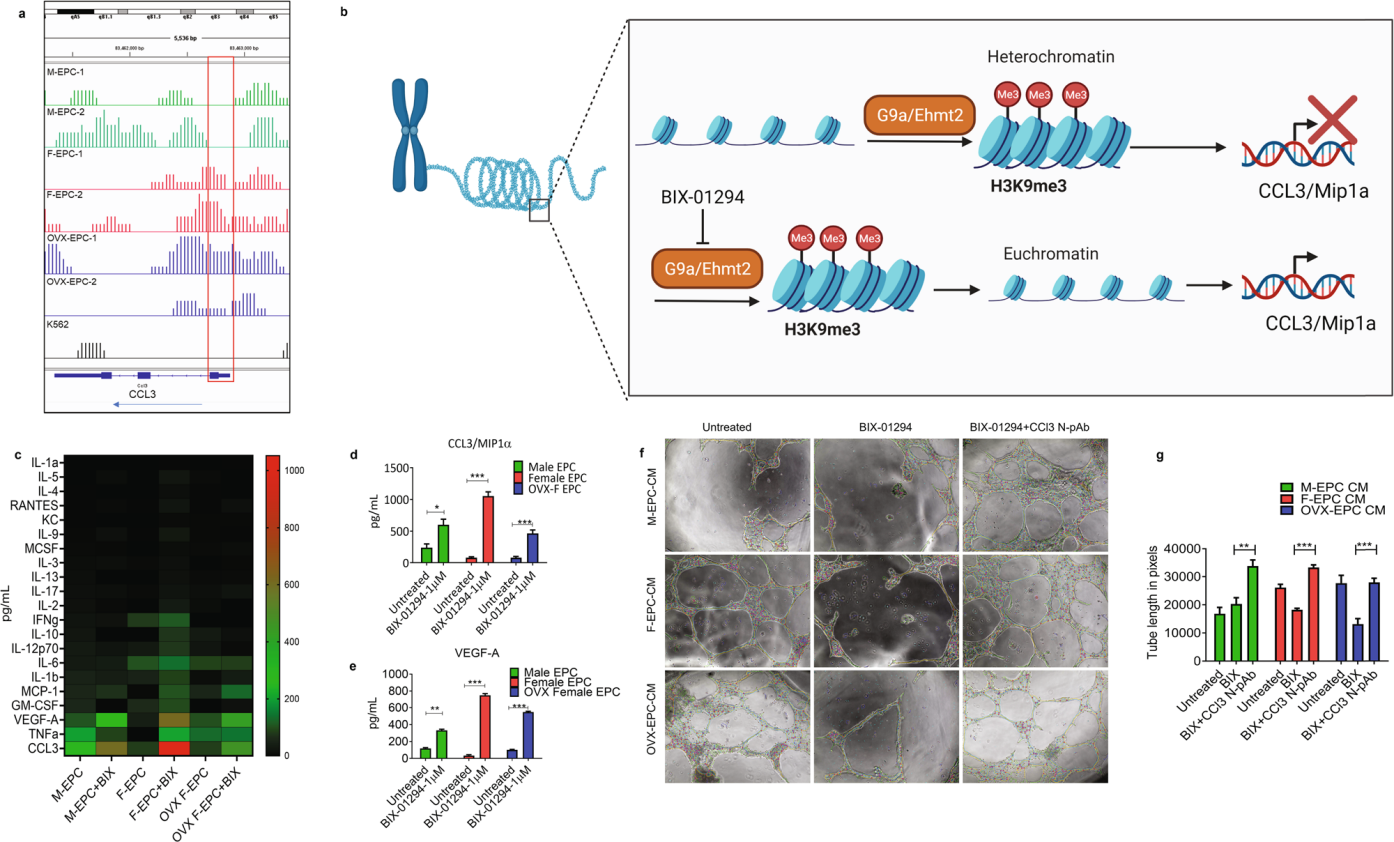
Inhibition of G9a/Ehmt2 using BIX-01294 regulates inflammation and angiogenic factors expression. **a** IGV screenshot of H3K9me3 specific peaks at the CCL3 gene region in M-EPCs, F-EPCs and OVX-EPCs (*n* = 2). The red box shows methylation peaks at the transcription start sites (TSS) of F-EPCs and OVX EPCs, and no methylation in the TSS region of M-EPC and K562 cells (positive control). **b** Schematic showing BIX-01294 dependent inhibition of G9a allows open chromatin to upregulate gene expression of CCL3. **c** Heat map of ELISA panel showing secreted inflammatory cytokines in M-EPC, F-EPC, and OVX EPC; CCL3 was found to be the most altered cytokine by inhibiting G9a using BIX-01294 (1 μM). Inhibition of G9a using BIX-01294 (1 μM) largely altered the secretion in the F-EPC and OVX EPC. ELISA showing secretory upregulation of CCL3 (**d**) and VEGF-A (**e**) in M-EPC, F-EPC, and OVX EPC with the inhibition of G9a using BIX-01294. **f**, **g** CM was collected from the sex differential EPCs pre and post-inhibition of G9a using BIX-01294. The CM was used to perform the tube formation assay using MCECs. CM containing high quantities of CCL3 was detrimental to tube formation, even in the presence of high VEGF quantities. Inhibition of CCL3 in the CM using a CCL3 neutralizing polyclonal antibody (CCL3-NpAb), resulted in robust tube formation indicating that CCL3 is an inhibitor of angiogenesis. *P < 0.01; **P < 0.001; ***P < 0.0005; ****P < 0.0001. Data are shown as mean ± s.e.m.

After getting a sex differential genome-wide snapshot of H3K9me3 occupancy (Fig. 6), we checked the H3K9me3 methylation status at the CCL3 gene and found high amounts of methylation in the TSS/promoter region of F-EPC and OVX EPC on the CCL3 gene indicating gene inactivation (Fig. 7a). In contrast, we did not see any H3K9me3 occupancy at the TSS of M-EPCs (Fig. 7a), reflecting their CCL3 expression and secretion (Fig. 2i). In addition, we investigated H3K9me3 occupancy in some of the key angiogenic gene regions such as Angpt-1, VEGFR2 and eNOS (Supplementary Fig. 8). We found higher H3K9me3 occupancy at the TSS of Angpt-1 (Supplementary Fig. 8a) as well as VEGFR2 (Supplementary Fig. 8c) in M-EPCs but not in F-EPCs and OVX-EPCs. Higher H3K9me3 occupancy at the TSS of Angpt-1 and VEGFR2 gene regions in M-EPC was reflected in their significantly lower gene expression in M-EPC compared with high expression on F-EPCs and OVX-EPCs (Supplementary Fig. 8b, d). Although no H3K9me3 occupancy was observed in the eNOS gene region, its gene expression in M-EPCs was lower compared with F-EPCs and OVX-EPCs, likely due to the higher expression of angiogenic ligands and receptors in female EPCs. It has been established that G9a/Ehmt2 histone methyltransferase catalyzes H3K9 trimethylation^15,35^. Therefore, we hypothesized that inhibiting G9a in EPCs using a small molecule inhibitor, BIX-01294, could inhibit G9a, reduce H3K9me3 methylation of several inflammatory genes, and enhance their secretion (Fig. 7b). The inhibition of G9a by BIX-01294 dramatically altered the secretome of M-EPCs, F-EPCs and OVX-EPCs, with varying magnitude (Fig. 7c). Although the levels of most proinflammatory factors such as CCL2, CCL5, IL-1β, IFNγ, TNFα, etc., increased, the quantities of CCL3 increased with the highest magnitude, particularly in F-EPCs and OVX-EPCs compared with M-EPCs explaining the tighter control of CCL3 by G9a induced H3K9me3 in female EPCs irrespective of estrogen influence (Fig. 7c, d). Although the levels of proinflammatory factors significantly increased in M-EPCs, the fold change of CCL3 secretion (Fig. 7d) was lower in M-EPCs compared with that of F-EPCs and OVX-EPCs (Fig. 7d). Female EPCs secreted the highest amounts of proinflammatory factors followed by OVX EPC and M-EPC with respect to fold change post-G9a inhibition by BIX-01294 (Fig. 7d). We observed a similar response in VEGF secretion upon BIX treatment (Fig. 7e). Using the CM from untreated and BIX-01294 treated sex dimorphic EPCs, we checked their in vitro angiogenic functionality (Fig. 7f, g). The CM from epigenetically modified EPCs with high amounts of CCL3 substantially inhibited in vitro angiogenesis despite the presence of proangiogenic factors. Inhibition of CCL3 in the CM using a neutralizing antibody specific to CCL3 significantly restored in vitro angiogenesis, likely promoted by VEGF, indicating that CCL3 is detrimental to angiogenesis (Fig. 7f, g). In addition, we used the CM from BIX-treated sex differential EPCs to evaluate the migration of monocytes in a scratch wound model system (Supplementary Fig. 9). CM from BIX-treated male, female, and OVX EPCs secreted high levels of CCL3 and promoted monocyte migration. Inhibition of CCL3 in CM from all types of EPCs significantly inhibited monocyte migration (Supplementary Fig. 9).

### Genetic modification of sex differential EPCs to evaluate loss and gain of function

**Fig. 8:**
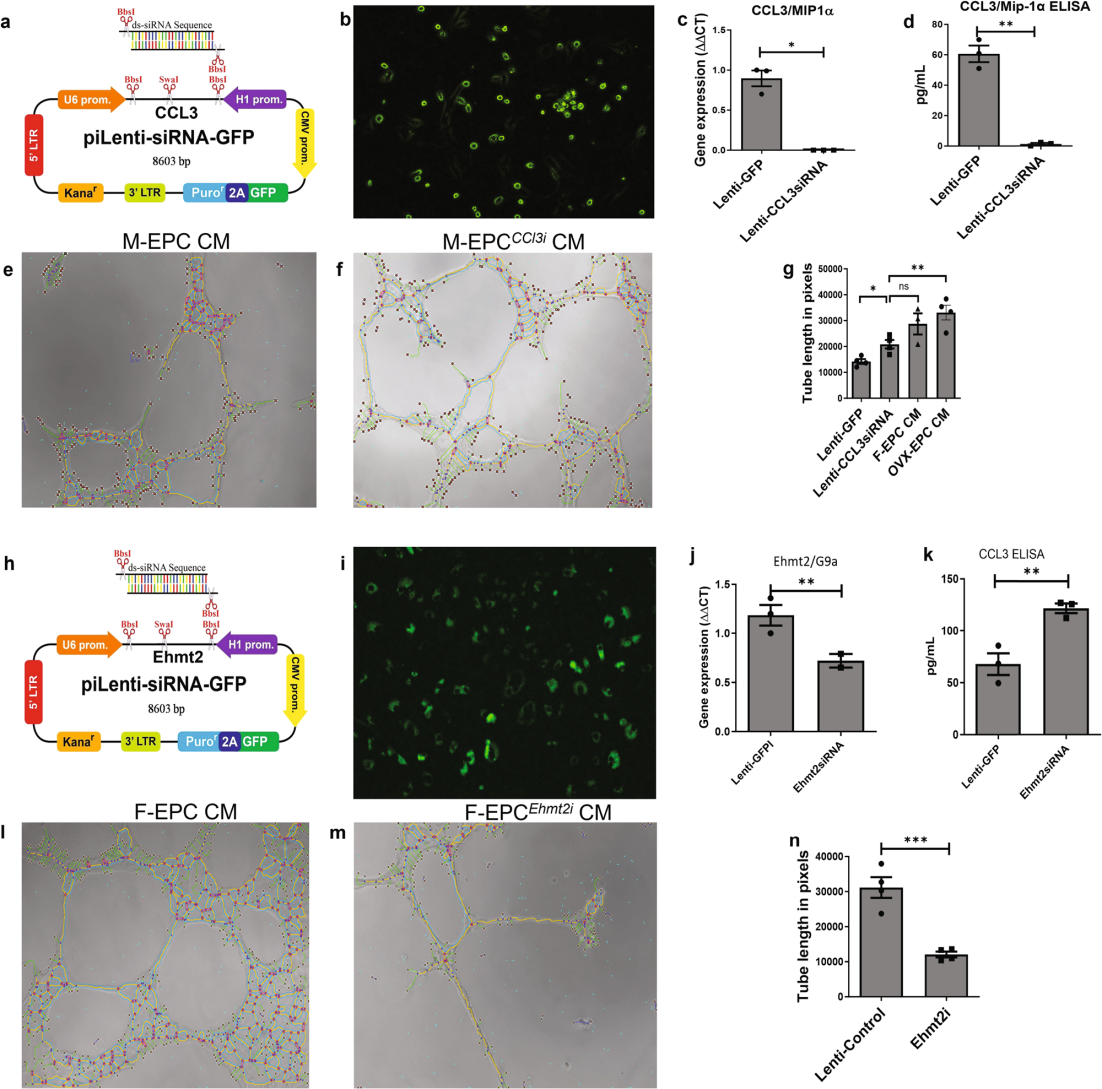
Genetic modification of EPCs to rescue phenotype and evaluation of G9a inhibition in F-EPCs. **a** Sequence map of CCL3-specific piLenti-siRNA. **b** confirmation of transfection and inhibition of CCL3 in M-EPCs by GFP expression post 72 hours. siRNA-based inhibition of CCL3 abrogated CCL3 gene expression (**c**) and secretion (**d**) in M-EPCs. **e** CM from M-EPCs, containing secreted CCL3, impaired tube formation. **f**, CM from CCL3-inhibited M-EPCs significantly restored tube formation. **g** Tube length estimation showing that CM from CCL3 inhibited M-EPCs rescued the phenotype to the level of F-EPCs. **h** Sequence map of G9a/Ehmt2-specific piLenti-siRNA. **i** GFP expression in Ehmt2/G9a inhibited F-EPCs. **j** pi-lenti-Ehmt2siRNA significantly reduced Ehmt2 gene expression (*P = 0.04). **k** Inhibition of Ehmt2 resulted in significant upregulation in the levels of CCL3 in F-EPC (**P = 0.0091). **l** F-EPC CM with low levels of CCL3 promoted efficient tube formation. **m**, **n**, CM from Ehmt2-inhibited F-EPCs, having high levels of CCL3, significantly (***P = 0.0008) inhibited tube formation of MCECs compared with Lenti-control F-EPCs; 1000× magnification. Data are shown as mean ± s.e.m.

Our next approach was to therapeutically improve the paracrine secretome of the male EPC phenotype to reduce its inflammatory effects as well as enhance its proangiogenic potential. Therefore, we first inhibited CCL3 in M-EPCs using lentiviral siRNA (Fig. 8a). After confirming CCL3 inhibition by GFP expression (Fig. 8b), qPCR (Fig. 8c) and ELISA (Fig. 8d), we used the CM from the CCL3 knockdown (KD) M-EPCs to evaluate their in vitro angiogenic function. We observed that CM from CCL3-KD M-EPCs promoted significantly higher tube formation of MCECs compared with Lenti-GFP control M-EPCs (Fig. 8e–g). Interestingly, the tube formation efficiency of CCL3-KD M-EPC CM was rescued to the levels of F-EPC CM (Fig. 8g). Next, we genetically modified the expression of Ehmt2 (G9a) in F-EPCs using an Ehmt2-specific siRNA (Fig. 8h, i). The Ehmt2-siRNA significantly reduced the expression of Ehmt2 in F-EPCs (Fig. 8j). Reduction in the expression of Ehmt2 resulted in significant upregulation in the secretion of CCL3 by F-EPCs (Fig. 8k). Further, Ehmt2-downregulated F-EPC CM showed significantly lesser tube formation (Fig. 8m, n) of MCECs compared with the piLenti-control F-EPC CM (Fig. 8l, n). Overexpression of Ehmt2 in both M-EPCs and F-EPCs had divergent effects on the secretion of CCL3 and VEGF. Ehmt2 overexpression in both male and female EPCs resulted in a reduction in the secretion of VEGF, while there was a concurrent increase in CCL3 secretion (Supplementary Figure 10a-j). Evaluation of CM from Ehmt2-overexpressed M-EPCs (M-EPC^*Ehmt2-OE*^) and F-EPCs (F-EPC^*Ehmt2-OE*^) (Supplementary Fig. 10a–j), resulted in impaired endothelial tube formation. This effect was likely a result of the diminished VEGF levels and the heightened CCL3 levels in the CM (Supplementary Fig. 10a–j).

To further confirm the detrimental effects of CCL3 in angiogenesis, we added recombinant CCL3 in the CM of F-EPCs and OVX-EPCs and performed MCEC tube formation experiments (Supplementary Fig. 10k–s). We found that the addition of CCL3 to the CM from F-EPCs and OVX-EPCs severely impaired tube formation compared with the CM controls (Supplementary Fig. 10k–s). The addition of CCL3 in complete EGM with fresh proangiogenic factors resulted in the abrogation of tube formation, reaffirming the role of CCL3 as an anti-angiogenic factor (Supplementary Fig. 10k–s).

## Discussion

The major finding in our study is that the superior cardiac reparative properties of female EPCs in promoting cardiac repair and remodeling rely on epigenetic mechanisms rather than the influence of sex hormones. Numerous studies have previously reported the role of sex hormones, especially estrogen and its receptors, in enhancing the cardio-protective properties of adult stem cells^4,36–38^. Conversely, sex hormone-independent aspects of sex differences have been insufficiently studied. Evidence exists that sex hormone-independent mechanisms may also affect disease disparity between the sexes. For example, sex-hormone-independent differences in stroke have been reported in pediatric patients and experimental animal models^39^. In animal models where sex hormone levels were similar, significant differences in neuroprotection were observed between the sexes^40^. However, not much has been studied to scrutinize sex differential epigenetic mechanisms of adult stem cells in cardiovascular disease regardless of sex hormone influences. Clinical studies have found higher EPC mobilization into the systemic circulation in fertile women than in postmenopausal women and age-matched men^41^. Clonogenic differences have also been found between female rat EPCs and male EPCs^41^. However, the higher number of EPC in the circulation of females, especially during menstrual cycles, is likely to support endometrial homeostasis^42^. Transcriptomic analysis of female and male muscle biopsies showed that female cells were enriched with genes associated with oxidative metabolism and protein catabolic pathways, which promote longevity and proliferation^26^. While these studies provide suggestive evidence, a direct comparison of sex-specific endothelial progenitor cells (EPCs) and other stem cells regarding their myocardial ischemic repair mechanisms, especially those unaffected by estrogen or other sex hormones, remains unexplored.

Limited available studies for direct comparison of functionality of sex-specific stem/progenitor cells prompted us to directly investigate sex differences in the cardiac reparative activity of EPCs^41,43,44^. Specifically, we also used EPCs from OVX mice to identify further sex differences in EPC function beyond the known effects of female sex hormones. Whole-genome mRNA transcriptomics showed that compared with F-EPCs and OVX-EPCs, M-EPCs showed a more proinflammatory gene expression pattern and were enriched in inflammatory genes such as IFNγ, CXCL9, IL1β, C3, CCL8, etc. which have been implicated in the progression of pathogenesis in heart diseases^45–47^. Conversely, both F-EPCs and OVX-EPCs showed upregulated expression of key angiogenic factors and receptors such as Angpt-1 and VEGFR2/Kdr. Angpt-1 is known to have powerful effects on Tie-2 expressing endothelial cells by enhancing their survival and preventing plasma leakage by stabilizing the integrity of vasculature^48^. Similarly, it has been well established that VEGFR2+ EPCs promote reendothelialization and neoangiogenesis to restore blood flow in the ischemic limbs of humans and mice^49^. In our experiments, proangiogenic factors secreted by F-EPC and OVX EPC efficiently promoted in vitro angiogenesis of MCECs compared with M-EPC. Further, the in vivo therapeutic evaluation of the sex differential EPCs in a mouse MI model revealed the superiority of both F-EPC and OVX EPC in protecting vasculature and promoting angiogenesis compared with M-EPC. Transplantation of M-EPC resulted in substantially high infiltration of inflammatory cells into the ischemic myocardium, which could be attributed to their increased secretion of proinflammatory cytokines. Moreover, we found significantly low numbers of anti-inflammatory/pro-reparative CD206^high^ M2-like cells in the M-EPC-injected group compared with female EPCs and vehicle recipient groups, indicating the detrimental effect of proinflammatory cytokines secreted by M-EPCs. Remarkably less infiltration of leukocytes was seen in groups of mice transplanted with F-EPC and OVX EPC. We anticipated that these differences in infiltrating cells were most likely due to CCL3, as it is secreted exclusively by the M-EPC compared with F-EPCs and OVX-EPCs. We further found that conditioned medium from M-EPCs with high levels of CCL3 promoted the migration of monocytes in vitro. Upon inhibition of CCL3 in M-EPC CM, ≥90% of monocyte migration was abrogated, confirming CCL3 as a major contributing factor of immune cell infiltration. CCL3 is also known to be a potent activator of adaptive and immune responses^50^. It is known to recruit most immune cells, including but not limited to monocytes, macrophages, T cells, neutrophils, and dendritic cells^50^. It was reported that inducing viral infection in CCL3^−/−^ mice significantly reduced the recruitment of CD8^+^T cells^51^. CCL3 and its receptor CCR5 have been shown to reduce osteocalcin, causing osteoblast dysfunction while enhancing pathogenesis in myeloma-induced bone disease^52^. CCL3 is also a stem cell inhibitor, unlike other cytokines such as CCL2 and CCL5^53^. Interestingly, CCL3 induces the differentiation of hematopoietic stem cells to myeloid cells, ultimately influencing the hematopoietic stem cell pool^54^. We further anticipated that high levels of inflammatory cytokines, including CCL3 secreted by male EPCs, could be due to underlying epigenetic modifications and unrelated to the estrogen status of EPCs.

Although research linking epigenetics and CVD is climbing, studies comparing sex-specific epigenetic regulations are scant. Epigenetic mechanisms have been described in X-chromosome inactivation in women^55^ for the control of sex-specific gene expression during development^56^ and in setting the stage for disease later in life^56^. Sex hormones have been shown to affect epigenetic modifications^57,58^. Sex has been considered a predictor of DNA methylation and gene silencing of histones at specific lysine or arginine residues, and different methylation patterns between female and male-derived cells/tissues have been reported^59–63^. However, whether sex alters histone or DNA methylation patterns in male and female EPCs or other stem cells is unknown. The contribution of other epigenetic mechanisms, such as posttranslational histone modifications and the role of non-coding RNAs as epigenetic modifiers, is not well studied in the context of sex differences. Whether sex-specific epigenetic mechanisms are involved in the differential functional properties of EPCs or other stem cells has never been studied. In the current study, we identified H3K9me3 specific whole epigenome methylation patterns as one of the epigenetic marks that govern the differential gene pattern and anti-inflammatory and pro-reparative properties of male, female, and OVX female EPCs. It has been established that the genomic occupancy of di- or trimethylation of histone 3 at lysine 9 (H3K9) at TSS or promoter gene regions is associated with gene silencing. Most of the H3K9me2/3 gene silencing is associated with embryonic, adult, or sex-related tissue homeostasis, differentiation, or maintenance^64^. SET-domain histone methyltransferases such as G9a and GLP were found to promote the catalysis of methylation on H3K9^65^. We found that genome-wide H3K9me3 occupancy was the highest in the male EPCs compared with female and OVX female EPCs indicating stark epigenomic variability between sexes. Upon investigating H3K9me3 at the CCL3 gene of sex dimorphic EPCs, we found high H3K9me3 occupancy at the TSS in F-EPC and OVX EPC and no methylation in the TSS of the M-EPC contributing to enhanced secretion of CCL3 by M-EPC resulting in augmented pathogenesis when injected in post-MI mice. Notably, we also found H3K9me3 occupancy in the TSS of essential angiogenesis-related genes such as Angpt-1, and VEGFR2/Kdr of M-EPCs, specifically at TSS/promoters causing reduced expression in M-EPCs (Supplementary Fig. 8). On the contrary, both F-EPCs and OVX-EPCs were unoccupied by H3K9me3 at the TSS of these angiogenic genes. In addition, we also observed that no H3K9me3 occupancy was found at the TSS in male, female, or OVX EPCs in the eNOS gene, a downstream molecule of the VEGF pathway that promotes angiogenesis. However, gene expression of eNOS was found to be significantly lower in M-EPCs compared with F-EPCs and OVX-EPCs, which could be attributed to H3K9 methylation and lower expression of VEGFR2 in M-EPCs (Supplementary Fig. 8). G9a/Ehmt2 histone methyltransferase (HMT) has been well reported to be involved in the catalysis of H3K9 di and tri-methylation in vitro and in vivo^65–67^. We show that inhibition of G9a using BIX-01294 as well as by G9a-specific siRNA resulted in the upregulation of CCL3 in all EPCs. A higher change in CCL3 secretion was observed in F-EPCs and OVX-EPCs, indicating higher methylation control of H3K9 at the TSS by G9a. Importantly, we found that higher secretion of CCL3 by EPCs is detrimental to angiogenesis. Inhibition of CCL3 in the secretome of BIX-treated EPCs, with high levels of CCL3, resulted in significant facilitation of angiogenesis, suggesting the anti-angiogenic effect of CCL3. To further confirm the anti-angiogenic activity of CCL3, we spiked CCL3 in the secretome of F-EPCs and OVX-EPCs lacking CCL3 and assessed tube formation, which resulted in significant abrogation of angiogenesis. Overexpression of G9a/Ehmt2 in M-EPCs and F-EPCs resulted in lower secretion of VEGF. However, CCL3 secretion was upregulated. This unexpected rise in CCL3 secretion prompted our speculation that it might be associated with a compensatory upregulation of histone demethylase, probably JMJD1. Our laboratory is actively investigating this intriguing possibility. This speculation suggests a potential interplay between G9a and JMJD1, suggesting further intricate regulatory mechanisms within the epigenetic landscape. Further exploration of the relationship between G9a and JMJD1 could yield valuable insights into the molecular pathways influencing CCL3 secretion, contributing to a more comprehensive understanding of epigenetic modulation in EPCs. A limitation of our current study is that we focused on H3K9me3 as the epigenetic mark associated with the differential gene expression pattern of sex-dimorphic EPCs. The contribution of other epigenetic mechanisms, such as DNA methylation, other posttranslational histone modifications, and non-coding RNAs, was not explored. While the study emphasizes epigenetic mechanisms unaffected by hormones, we acknowledge that sex hormones may still exert organizational effects on genomic and epigenomic mechanisms. The investigation could benefit from a more comprehensive exploration of hormonal influences on epigenetic regulation.

Based on the study results, we infer that epigenetic mechanisms, specifically those associated with genomic occupancy of H3K9me3, significantly influence specific cardiac reparative properties of adult bone marrow stem cells. Mechanistically, we show that these sex-based functional differences of bone marrow stem cells are mediated, in part, by differential epigenetic regulation of chemokine CCL3.

## Methods

### Cell culture and treatments

EPCs from male, female, and OVX mice were isolated following previously established methods from our laboratory^68^ with certain modifications. Briefly, bone marrow cells were harvested and fractionated using Histopaque -1083 (10831, Sigma-Aldrich, USA), and the monocyte layer was subjected to multi-sort magnetic-activated cell sorting (Miltenyi Biotec, Germany) as per the manufacturer’s instructions. Lineage cells were first separated using the Lineage Cell Depletion kit (130-110-470, Miltenyi Biotec, Germany). Further, an Anti-FITC multisort kit (130-058-701, Miltenyi Biotec, 10 μl/10^7^ cells, Germany) was used to primarily isolate Sca-1 cells tagged with anti-Sca-1-FITC antibody (11-5981-82, 0.5 μg/test, ThermoFisher, USA). After the separation of Sca-1^+^ cells, the primary antibodies with magnetic beads were released, tagged with CD31 microbeads (130-097-418, 10 μl/10^7^ cells, Miltenyi Biotec, Germany), and further subjected to magnetic separation to obtain Sca-1^+^/CD31^+^ EPC fraction. The cells were then plated and cultured using endothelial growth medium 2 (EGM-2; low phenol red; no estrogen, Lonza, Switzerland) on 0.2% gelatin-coated plates (Primaria^TM^ 6-well plates, Corning®, USA). For some assays, BIX-01294 (1 μM, HY-10587, MedChemExpress, USA) was used to treat EPCs for 12–16 h followed by changing the medium for collecting conditioned medium. Human umbilical vein endothelial cells (HUVEC) or mouse cardiac endothelial cells (MCEC) were purchased from an American-type culture collection (ATCC, USA) and cultured using EGM-2. The Raw 264.7 monocytes were cultured in DMEM-F12 medium (Corning, USA) with 10% fetal bovine serum (FBS) and 1× penstrep^TM^.

### Flow cytometry

Flow cytometry was employed to characterize and identify the percentage of CD31/Sca-1 cells in the isolated EPCs, prior to specific magnetic sorting. Male, female, and OVX-EPCs were incubated with Sca-1-FITC (Cat no. 11-5981-81, 0.5 μg/test, ThermoFisher, USA) and CD31-PE (Cat no. 25-0311-820, 5 μg/test, ThermoFisher, USA) antibodies for 30 minutes in room temperature. Appropriate compensation controls were used to negate spectral overlap. Samples were analyzed using the BD FACSAria^TM^ (BD Biosciences, USA) and FACSDiva^TM^.

### RNA extraction, reverse transcription, and RT-PCR

RNA was isolated from cells using miRNeasy Mini kit (Qiagen, Germany, 1038703). NanoDrop-1000 (Thermo Scientific, USA) was used to identify RNA concentration and purity determined by the 260 A/280 A ratio. High-capacity cDNA Reverse Transcription Kit (Applied Biosystems, 4368814) was used to obtain cDNA. RT-PCR was performed on an Applied Biosystems 770 StepOnePlus system using the Fast SYBR^TM^ Green Master Mix (Applied ThermoFisher, USA) according to the manufacturer’s instructions. Fold changes were normalized to GAPDH with the threshold delta-delta cycle method. PCR array kit was used to assess angiogenic and inflammatory genes (PAMM-024Z, Qiagen, Germany). Primer list in Supplementary Table 1.

### RNA sequencing

RNA quantity was evaluated using Qubit RNA HS assay (ThermoFisher, USA), and the quality was confirmed using Bioanalyzer 2100 Eukaryote Total RNA Nano (Agilent Technologies, USA). All samples had RNA integrity based on a RIN of >8. The RNA-seq libraries were prepared according to the protocol of Illumina mRNA-Seq TruSeq. DNA libraries were generated using NEBNext Ultra II Directional RNA prep kit (New England Biolabs, USA).1 μg of total RNA was the input, followed by PCR enrichment using NEBNext multiplex oligos for Illumina. DNA libraries were run on a NovaSeq 6000 sequencer (Illumina, USA). A total of 9 fastq files were generated (3 for M-EPCs, 3 for F-EPC, and 3 for OVX-EPCs). Data quality statistics were assessed and filtered clean reads were mapped to reference genome using HISAT2. Cuffquant and Cuffnorm components of Cufflinks software were used to quantify gene expression levels using mapped reads positional information on the gene. DEseq was used to analyze the differentially expressed genes (DEG) for samples with biological replicates. Fold change ≥2, and FDR > 0.05 were set as screening criteria. Gene enrichment analyses of upregulated and downregulated genes were performed using KEGG and GO databases.

### Conditioned medium collection and measurement of cytokines and growth factors

The conditioned medium (CM) was collected from the EPCs in endothelial basal medium (EBM- (without growth factors and with 5% FBS) for 72 h. The CM was analyzed for the presence of cytokines, chemokines, and growth factors by performing a semi-quantitative (Chemiluminescence, R&D Systems, USA) and quantitative mouse-specific antibody-based array (Quantibody array, RayBio®, USA). The experiment was performed as per the manufacturers’ instructions. The data recorded were analyzed using Image J software (NIH, USA). The relative intensities of individual growth factors were calculated as arbitrary units after background correction and normalized to control media blot intensities or relative fluorescent units (RFUs) correlated to each cytokine’s standard curves to get quantitative values.

#### Endothelial cell migration assay

The cell migration assay was performed using two-chamber cell Transwell inserts with an 8 μm-pore polycarbonate membrane (Corning, USA). HUVECs or MCECs were serum-starved for 18 h in the endothelial basal medium supplemented with 0.1% FBS. Then, the cells were trypsinized and reconstituted in the endothelial basal medium at a density of 1 × 10^5^ cells/100 μl. Next, 1 × 10^5^ cells/well were plated into the upper chamber of the Transwell insert. In the lower chamber, CM collected from the different EPCs was used at 50% dilution along with endothelial basal medium, endothelial growth medium (with 10% fetal bovine serum—without growth factors), and endothelial growth medium (complete) controls. HUVECs/MCECs were allowed to migrate for 18 h at 37 °C in a humidified incubator. The non-migrated cells were carefully removed using a pre-wet cotton swab, following which the membrane was fixed with 4% paraformaldehyde (Sigma-Aldrich, USA) and stained with DAPI for 5 minutes. After washing, the membrane was mounted on a slide with DPX (Sigma-Aldrich, USA; cat no. 44581), and the cells that migrated from the upper to the lower side of the membrane were counted manually from 10 random field images taken per condition under the Eclipse Ti fluorescence microscope (Nikon, Japan) using 20× objective. All the assays were performed in duplicates. The data were represented as mean ± SEM.

### Tube formation assay

Growth factor reduced (GFR) matrigel (Corning®, USA) was thawed at 4 °C overnight on ice. Exactly 10 µl of matrigel was coated onto each inner well of the µ-angiogenesis slides (IBIDI, Germany) and solidified at 37 °C for 30 min. A total of 1 × 10^4^ HUVECs were reconstituted in CM from the different EPCs, CM containing anti-Ccl3 neutralizing antibody (1 µg/mL), EGM or serum-free medium to a total volume of 50 µl and plated on the GFR matrigel. The plates were incubated for 6 h in a humidified incubator at 5% CO2 and 37 °C. Images were taken using an inverted phase-contrast microscope (Nikon, Japan) under 4× and 10× objectives. The tube length was measured using WimTube (Wimasis, GmbH, Germany) from the 1000× magnification images of 3 wells in each condition. The experimental samples and controls were assayed in duplicates.

### Monocyte migration assay

The Raw 264.7 monocytes were plated in 24 well plates and allowed to grow to confluence. The monocytes were serum starved with DMEM + 0.1% FBS upon confluency for 8 hours. The scratch wound was induced using a 100 μl tip. Images were taken at the start of the study, and then the different conditions were added, which included M-EPC, F-EPC, and OVX-EPC CM with or without CCL3-N-pAb. Plain DMEM and DMEM with serum were used as controls.

### CUT&Tag-IT^TM^ sequencing assay

The Sca-1^+^/CD31^+^ male (M), female (F), and OVX EPC were harvested using a non-enzymatic method (cell scraping). Cells were washed with DPBS, counted, and 1 × 10^6^ cells per vial were frozen in a frozen mix (90% FBS + 10% Dimethyl sulfoxide-DMSO) and set aside in duplicates for CUT&Tag^TM^ sequencing. Approximately 2 × 10^5^ nuclei were used for performing CUT&Tag-IT^TM^ sequencing using a standard assay kit (Active Motif, USA cat no. 53160) for the H3K9me3 mark using a CHIP grade antibody (Cat no. 391691, Active Motif, USA). Further downstream steps were performed according to the protocol provided in the kit (Active Motif, 53160). DNA libraries were prepared using unique i7 and i5 index primers for each sample, size selected with SPRI beads (Cat No. 53160, Active Motif), quality checked with fragment analyzer, a capillary electrophoresis instrument for NGS library quality check and sequenced by the UMass Deep Sequencing Core with 25 bp paired-end Illumina Miseq system. Paired-end reads were trimmed to 25 bases, barcodes were removed, and reads were aligned to mm10 using Bowtie2 with the parameters -N 1 and -X 1000. Duplicates were removed using Picard. Low-quality score reads (MAPQ < 10) were removed. These reads were processed in HOMER. Genome browser tracks were generated from mapped reads using the “makeUCSCfile” command. Mapped reads were aligned using the “annotatePeaks” command. To identify regions of the genome that exhibited differential occupancy, we used the ChIPseeker Bioconductor package in the DolphinNext platform. As input files, we used called peaks from MACS analysis.

### Animals

All animal procedures were performed following the approved protocols of the Institutional Animal Care and Use Committee (IACUC) of Temple University. Eight-week-old male, female, or ovariectomized female (OVX) C57BL/6 J mice were purchased from Jackson Research Laboratory (JAX) (Bar Harbor, ME) for EPC isolation. For the OVX mice, ovariectomy is performed by JAX at around 35 days of age, and they are sent to our facility at 8 weeks of age. The levels of 17 β Estradiol were measured to confirm successful ovariectomy. The mice 10–12 weeks old male C57BL/6 J mice were used for MI surgery.

### MI Surgery

Mice were subjected to MI by ligating the LAD coronary artery as described previously (17). Mice were anesthetized by 2–3% isoflurane and orally intubated with a 22 G i.v. catheter, and artificially ventilated with a respirator (Harvard Apparatus). To provide analgesia, buprenorphine SR (0.5 mg/kg) was injected s.c. before the operation. A left intercostal thoracotomy was performed, and the ribs were retracted with 5-0 polypropylene sutures to open the chest. After the pericardium was opened, the LAD branch of the left coronary artery was ligated distal to the bifurcation between the LAD and diagonal branch using 8-0 polypropylene sutures through a dissecting microscope. After positive end-expiratory pressure was applied to inflate the lung fully, the chest was closed with 7-0 polypropylene sutures. A 22 G syringe was used to evacuate air from the chest cavity. The survival rate of MI surgery was 87.5%. The mice in the sham group underwent the same procedure except for the LAD ligation. At the end-point, heart tissues were collected under anesthesia with Avertin® (222-Tribromoethanol; T48402; MilliporeSigma, USA). The animals were then euthanized following the procedure from our approved IACUC protocol.

### Echocardiography

Transthoracic two-dimensional M-mode echocardiography using the Vevo2100 equipped with 30 MHz transducers (VisualSonics, Toronto, ON, Canada) was performed before MI (baseline), and 1-, 2-, 3-, and 4- weeks after surgery as described previously. Mice were anesthetized with a mixture of 1.5% isoflurane and oxygen (1 L/min) with an isoflurane delivery system (Viking Medical, Medford, NJ). The internal diameter of the LV was measured in the short-axis view from M-mode recordings; percent ejection fraction (% EF) and fractional shortening (% FS) were calculated using corresponding formulas as previously described.

### Tissue preparation and immunohistochemistry

Mouse heart tissue samples were fixed in 10% formalin for at least 48 hours and embedded in paraffin. Cardiac tissues were cross-sectioned into 4–5 μm-thick slides. Masson Trichrome staining (Sigma Aldrich, USA) was performed following the manufacturer’s instructions and previously described in detail. For the identification of endothelial cells and pan-immune cells, CD31 (AF3628; 1:30 dilution, R&D Systems, USA), CD45 staining (AF114; 1:50 dilution, R&D Systems, USA), and CD206 (AF2535; 1:50 dilution, R&D Systems, USA;) were used, respectively. Donkey anti-goat (A-21432, ThermoFisher, USA) or donkey anti-rabbit (A-31572, ThermoFisher, USA) secondary antibodies were used at a dilution of 1:100. Images were acquired using the Eclipse Ti fluorescence microscope (Nikon, Japan) using 20x objective and planimetry analysis using ImageJ.

### Vectors for knockdown and overexpression

An iLenti RNAi lentivirus expression system with a pool of four target sequences with a green fluorescent protein (GFP) reporter was used for the knockdown of mouse CCL3 (153670940296, ABM) and Ehmt2 (190580940296, Abmgood, Canada) in cells. For overexpression, a CMV promoter-driven genes of interest (GOI) expression with a red fluorescent protein (RFP) reporter was used (CCL3—153670640496; Ehmt2—190580640495; Abmgood, Canada). EPCs were transfected with an MOI of 5 along with ViralEntry^TM^ transduction enhancer (G515, Abmgood, Canada) for 8 h in serum-free EGM media. After 8 h, the medium was changed, and gene expression was confirmed after 72 h.

### Statistical analysis

Statistical analyses were performed using GraphPad Prism 9.0 software (GraphPad, La Jolla, CA). All Data are presented as mean ± SEM and represent at least 3 independent biological experiments. An unpaired *t*-test was used to compare 2 sample groups. For comparisons of more than two groups, one-way ANOVA was done with a Tukey Posthoc test followed by Dunn’s pairwise comparisons. Two-way ANOVA with Tukey’s multiple comparisons test was used for echocardiography parameters with repeated measures over time. *P* value of <0.05 was considered statistically significant.

### Reporting summary

Further information on research design is available in the Nature Research Reporting Summary linked to this article.

### Supplementary information

Supplemental information

Reporting summary

## Data Availability

The datasets of RNA-seq for male, fertile female, and ovariectomized female EPC generated during this study are available at GEO: GSE253058. The datasets of Chip-seq (CUT&TAG) for H3K9me3 in male, fertile female, and ovariectomized female EPCs are available at GEO: GSE253057. All other data supporting the findings of this study are available from the corresponding author upon reasonable request.

## Code availability

In this work, only public software and custom code were used to generate or process datasets. The software version and websites were described in the “Methods” section.
