# Supplementary material for: Epigenetic mechanisms regulate sex differences in cardiac reparative functions of bone marrow progenitor cells
Source: NPJ Regen Med. 2024 Apr 29;9:17. doi: 10.1038/s41536-024-00362-2 (PMC11058271; doi:10.1038/s41536-024-00362-2)
Supplement: Supplementary file 1 — Supplemental information [file 41536_2024_362_MOESM1_ESM.pdf]

Supplementary Information

Epigenetic Mechanisms Regulate Sex Differences in Cardiac Reparative Functions of Bone Marrow Progenitor Cells

Charan Thej[1], Rajika Roy[4], Zhongjian Cheng[1], Venkata Naga Srikanth Garikipati[1], May M Truongcao[1], Darukeshwara Joladarashi[1], Vandana Mallaredy[1], Maria Cimini[1], Carolina Gonzalez[1], Ajit Magadum[1], Jayashri Ghosh[3], Cindy Benedict[1], Walter J. Koch[1,4], *Raj Kishore[1,2]

[1]Aging and Cardiovascular Discovery Center, Lewis Katz School of Medicine, Temple University, Philadelphia, PA, 19140. [2]Department of Cardiovascular Sciences, Lewis Katz School of Medicine, Temple University, Philadelphia, PA 19140, USA. [3]Fels Cancer Institute for Personalized Medicine, Lewis Katz School of Medicine, Temple University, Philadelphia, PA 19140, USA. [4]Department of Surgery, Division of Cardiovascular and Thoracic Surgery, Duke University School of Medicine, Durham, NC

**Corresponding Author: raj.kishore@temple.edu*

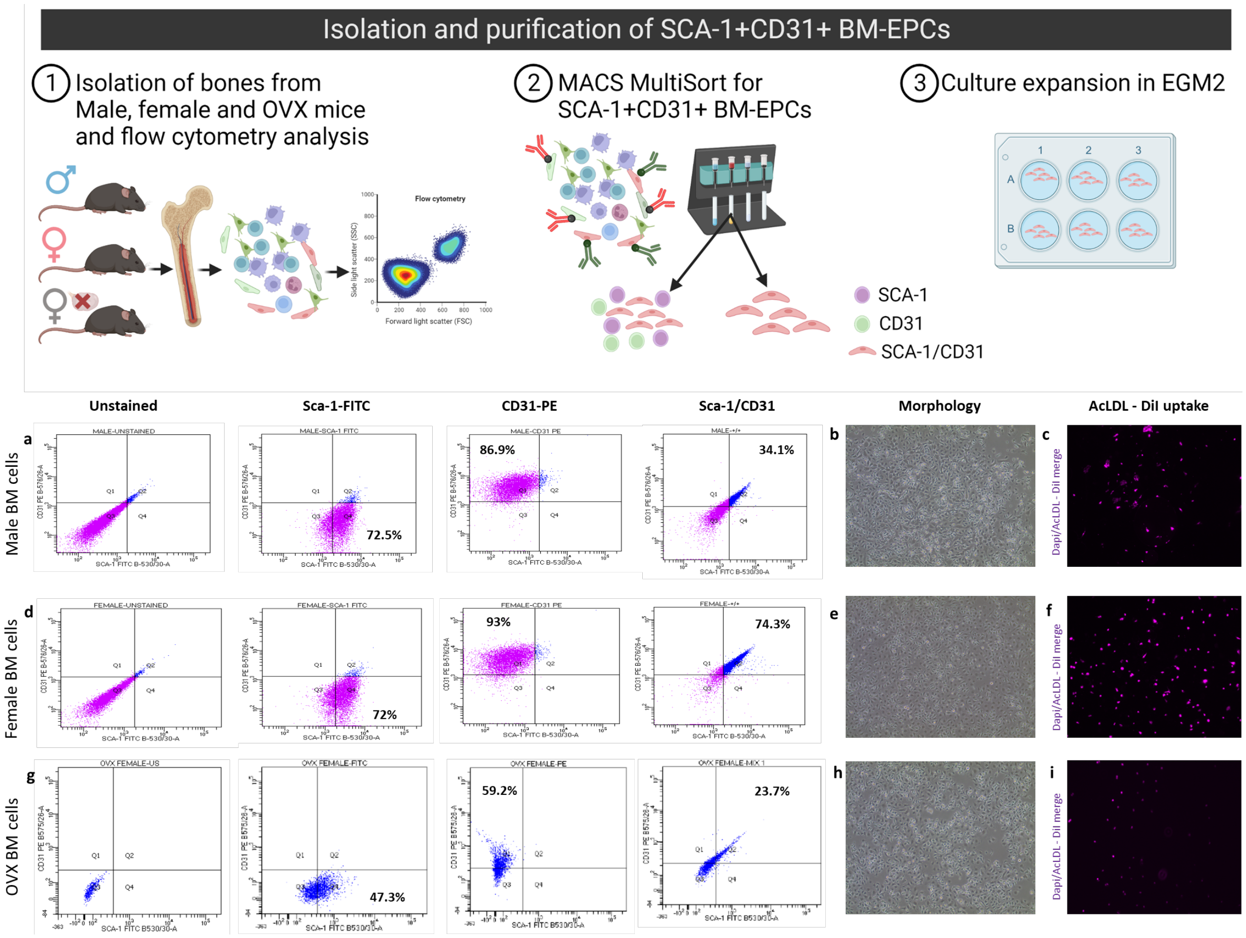

Supplementary Figure 1. Characterization of sex differential EPCs. **a, d & g**, Bone marrow cells from Male, female and OVX mice were evaluated for the presence of Sca-1+/CD31+ population using flow cytometry prior to MACS sorting. Post-sorting of Sca-1+/CD31+ cells` using multi-MACS method, and culturing to confluence in EGM-2 medium, EPCs were confirmed by morphological analysis (**b, e & h**) as well as uptake of acetylated-LDL-DiI (Ac-LDL-DiI) (**c, f & i**) to confirm endothelial phenotype. 1000× magnification.

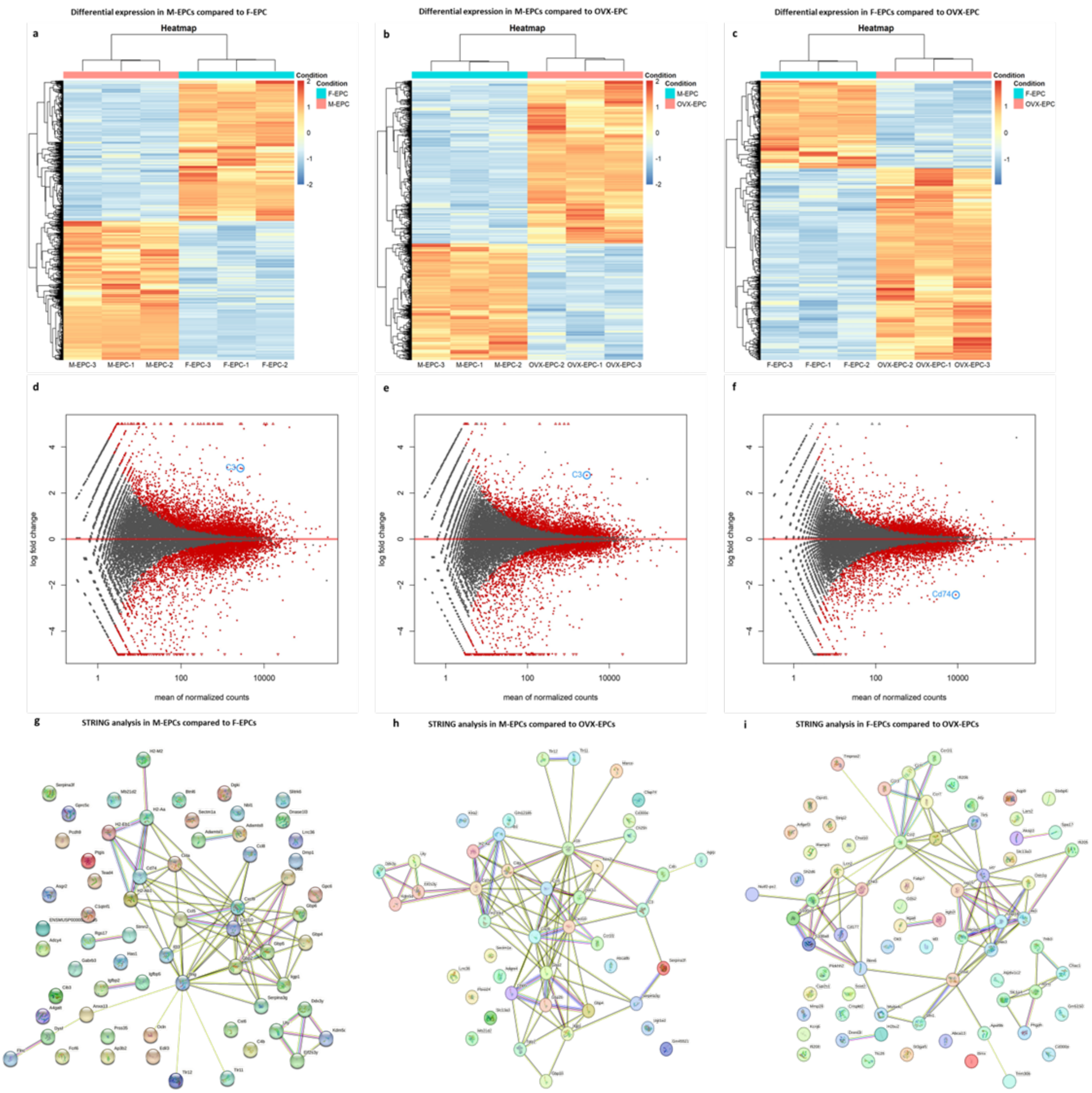

Supplementary Figure 2. **Whole transcriptome RNA sequencing of M-EPCs, F-EPCs and OVX-EPCs.** Heatmap showing differential expression between M-EPCs vs. F-EPCs (**a**), M-EPCs vs. OVX-EPCs (**b**) & F-EPCs vs. OVX-EPCs (**c**). MA plot representation of M-EPC vs. F-EPCs (**d**), M-EPCs vs. OVX-EPCs (**e**) & F-EPCs vs. OVX-EPCs (**f**) showing significantly higher differential gene expression between M-EPC vs. F-EPCs and M-EPCs

vs. OVX-EPCs with highly significant differential expression of C3, a proinflammatory marker. Fewer differences in gene expression were seen between F-EPCs vs. OVX-EPCs with top differential expression of CD74, an adhesion marker. STRING analysis in M-EPC (**g**) shows high interactions of proinflammatory cytokines and chemokines such as IFNγ, CCL5, CCL8, CXCL9, CXCL10, etc., compared with lower proinflammatory chemokine interactions in interactions in F-EPCs (**h**) and OVX-EPCs (**i**).

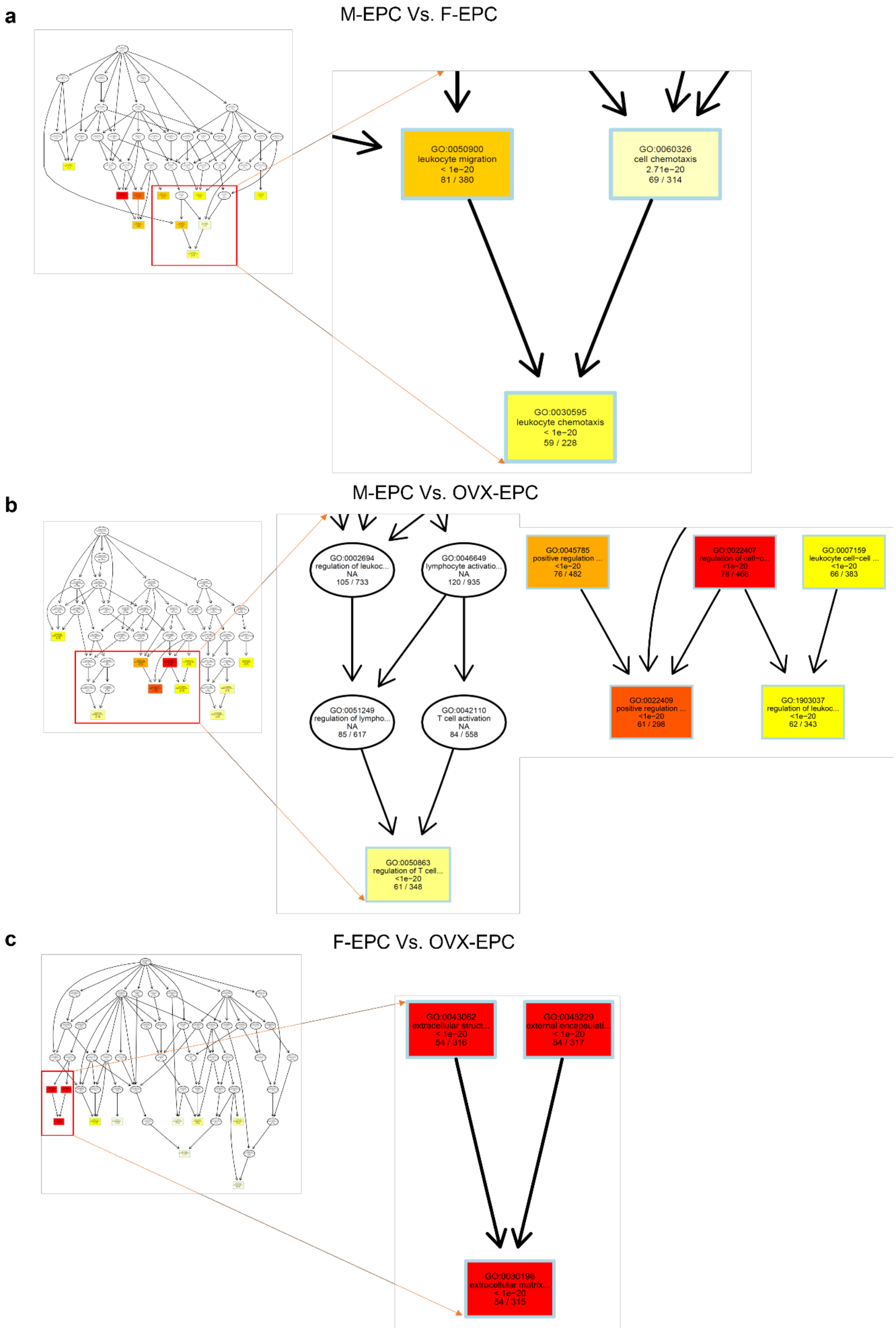

a
M-EPC Vs. F-EPC
GO:0050900
leukocyte migration
< 1e−20
81 / 380
GO:0060326
cell chemotaxis
2.71e−20
69 / 314
GO:0030595
leukocyte chemotaxis
< 1e−20
59 / 228
b
M-EPC Vs. OVX-EPC
GO:0002694
regulation of leukoc...
NA
105 / 733
GO:0046649
lymphocyte activatio...
NA
120 / 935
GO:0051249
regulation of lympho...
NA
85 / 617
GO:0042110
T cell activation
NA
84 / 558
GO:0050863
regulation of T cell...
<1e−20
61 / 348
GO:0045785
positive regulation ...
<1e−20
76 / 482
GO:0022407
regulation of cell−c...
<1e−20
78 / 468
GO:0007159
leukocyte cell−cell ...
<1e−20
66 / 383
GO:0022409
positive regulation ...
<1e−20
61 / 298
GO:1903037
regulation of leukoc...
<1e−20
62 / 343
c
F-EPC Vs. OVX-EPC
GO:0043062
extracellular struct...
< 1e−20
54 / 316
GO:0045229
external encapsulati...
< 1e−20
54 / 317
GO:0030198
extracellular matrix...
< 1e−20
54 / 315

Supplementary Figure 3. **Comparison of gene ontology of biological process in** M-EPCs vs. F-EPCs (**a**), M-EPCs vs. OVX-EPCs (**b**) & F-EPCs vs. OVX-EPCs (**c**). **a & b**, Compared with F-EPCs and OVX-EPCs, groups of genes associated with leukocyte migration, cell chemotaxis, leukocyte chemotaxis, positive regulation of immune response, regulation of T-cell proliferation were significantly upregulated in M-EPCs. **c**, In contrast, only extracellular matrix-associated genes were differentially expressed in F-EPCs compared with OVX-EPCs.

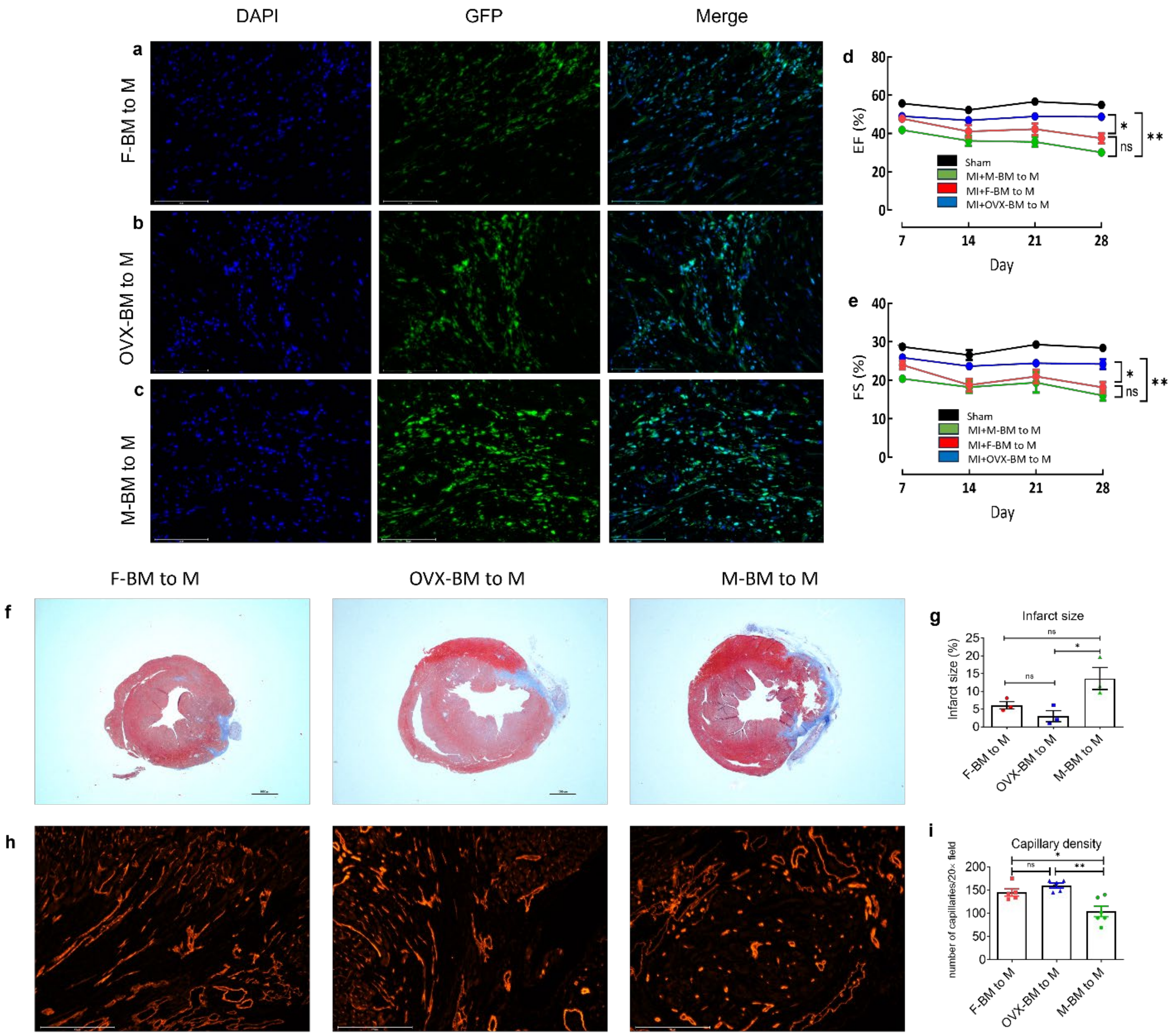

Supplementary Figure 4. Chimeric bone marrow transplantation and evaluation of cardiac repair post-MI. BM from donor male, female and OVX GFP transgenic mice were transplanted in recipient male mice following irradiation (5.5Gy). Mobilization of GFP+ cells to the ischemic heart 3 days post-MI in male mice reconstituted with male (**a**), female (**b**) and OVX (**c**) GFP+ bone marrow; scale bar=125μm. **d** & **e** Chimeric male mice harboring female and OVX-female BM showed improved post-MI %EF (P=0.09 vs F-EPC; **P=0.002 vs OVX-EPC) and %FS (P=0.2 vs F-EPC; **P=0.001). **f** & **g**, Masson's trichrome staining showed significantly higher infarct size in the chimeric male mice with male BM compared

with chimeric mice with female and OVX female BM; scale bar=125µm. **h**, CD31 staining shows significantly higher number of capillaries in 28 days post-MI hearts of chimeric mice with female and OVX female BM compared with male BM chimeric mice hearts; scale bar=275µm. *P<0.01; **P<0.001; ***P<0.0005; ****P<0.0001;. Data shown as mean ± s.e.m.

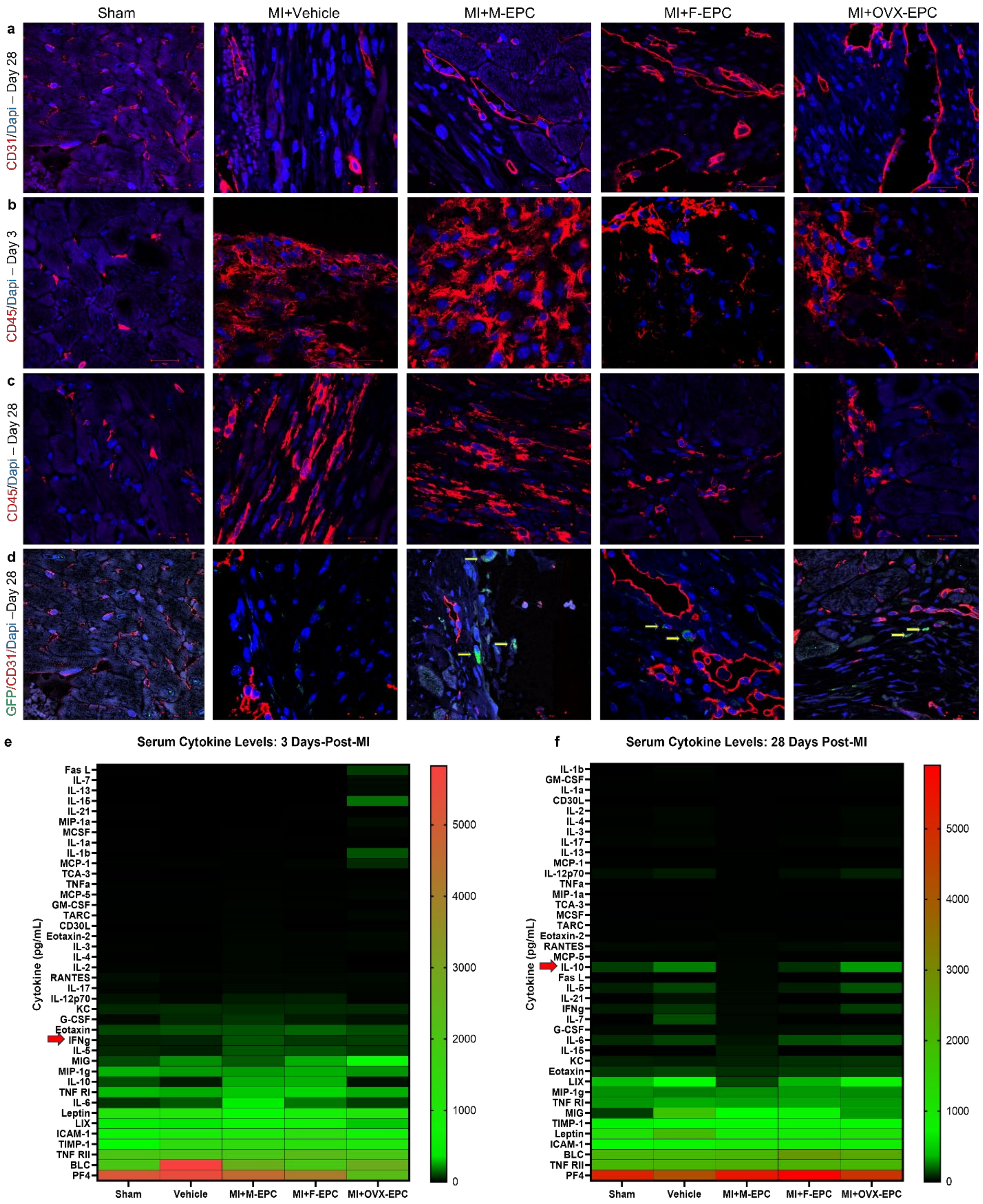

Supplementary Figure 5. ***In vivo* angiogenesis and inflammation. a,** confocal image of CD31 staining in day 28 sham/post-MI hearts from various treatment groups. **b,** confocal

image of CD45 staining in hearts of different groups at day3 (**b**) and day 28 (**c**). **d**, Male, female and OVX EPCs transfected with piLenti-GFP plasmid were injected into post-MI mice and were detected at Day 28 in the hearts, albeit in low numbers; scale bar=20μm. Heat map showing quantification of inflammatory factors in serum of various treatment groups collected at day 3 (**e**) and day 28 (**f**); scale bar=20μm.

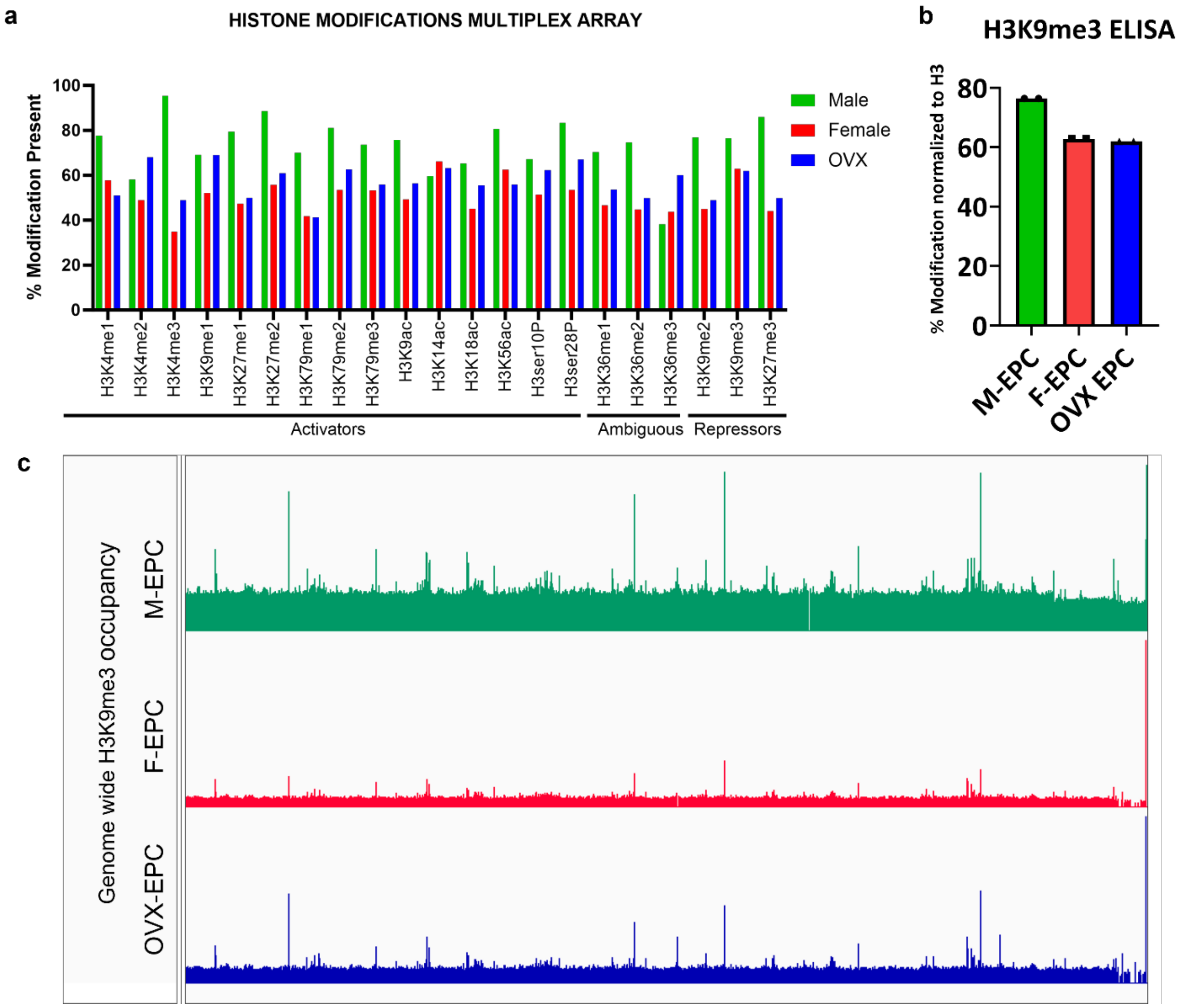

Supplementary Figure 6. Histone modifications in male, female and OVX EPCs. **a**, Multiplex ELISA array of histone modifications in M-EPC, F-EPC and OVX-EPCs. **b**, ELISA of H3K9me3 in M-EPCs, F-EPCs and OVX-EPCs. **c**, IGV browser screenshot of H3K9me3 occupancy in M-EPCs, F-EPCs and OVX-EPCs. *P<0.05; **P<0.001; ***P<0.0001.

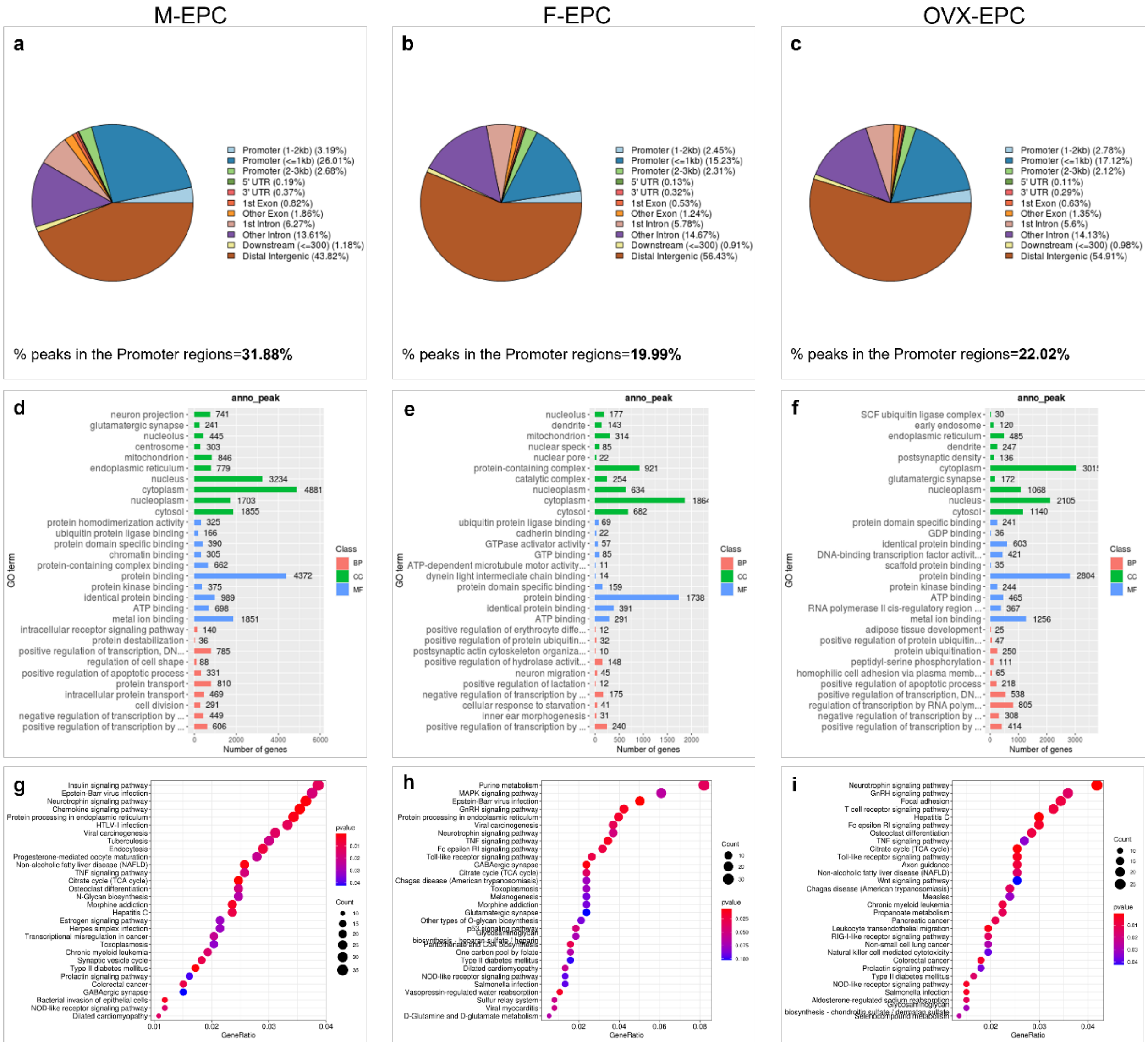

Supplementary Figure 7- CUT&Tag sequencing of M-EPC, F-EPC and OVX EPC. Pie-charts indicating H3K9me3 occupancy in different genomic regions. Higher H3K9me3 occupancy was observed in M-EPCs (31.88%) (**a**) compared with F-EPC (19.99%) (**b**) and OVX-EPC (22.02%) (**c**). Gene ontology of sex differential EPCs showing biological processes, cellular components and molecular functions associated with H3K9me3 specific methylation peak annotations showing high occupancy in M-EPCs (**d**) compared with F-EPCs (**e**) and OVX-EPCs (**f**). KEGG pathway analysis of H3K9me3 peak regions in male (**g**), female (**h**) and OVX EPCs (**i**). H3K9me3 occupancy was found to be significantly

higher at TNF signaling pathway genes in F-EPCs and OVX-EPCs. MAPK signaling genes and T cell receptor signaling pathway-associated gene regions were occupied with the H3K9me3 mark in F-EPCs and OVX-EPCs respectively.

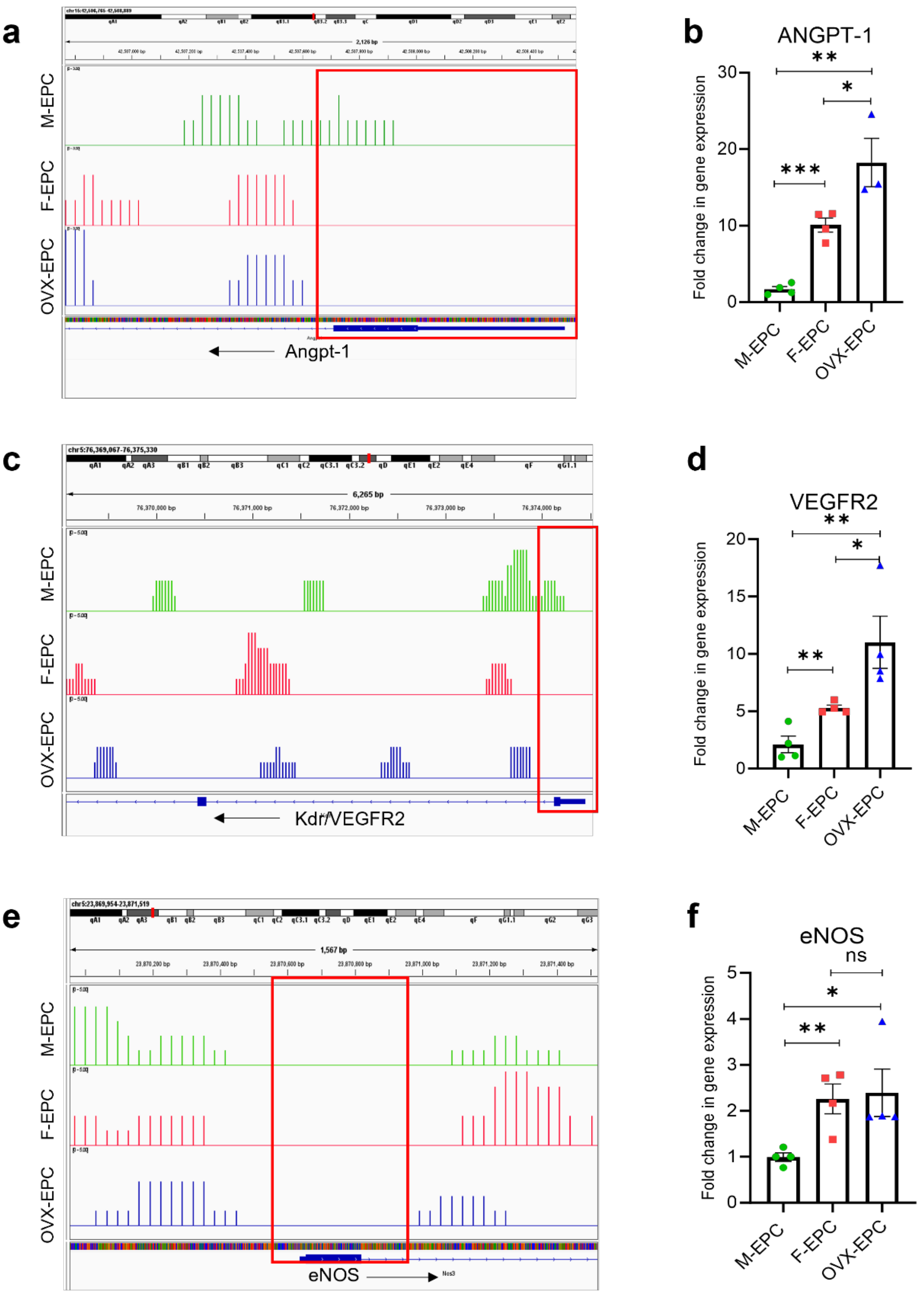

Supplementary Figure 8- H3K9me3 occupancy on proangiogenic genes. IGV browser screenshot of H3K9me3 occupancy on the genomic region of Angpt-1 (**a**), Tie-2/Tek (**b**), VEGF-A (**e**), VEGFR-2/Kdr (**f**), and eNOS/NOS3 (**i**) in M-EPCs, F-EPCs and OVX-EPCs.

Gene expression of Angpt-1 (**c**), Tie-2/Tek (**d**), VEGF-A (**g**), VEGFR-2/Kdr (**h**), and eNOS/NOS3 (**j**) in M-EPCs, F-EPCs and OVX-EPCs. *P<0.01; **P<0.001; ***P<0.0005. Data shown as mean ± s.e.m.

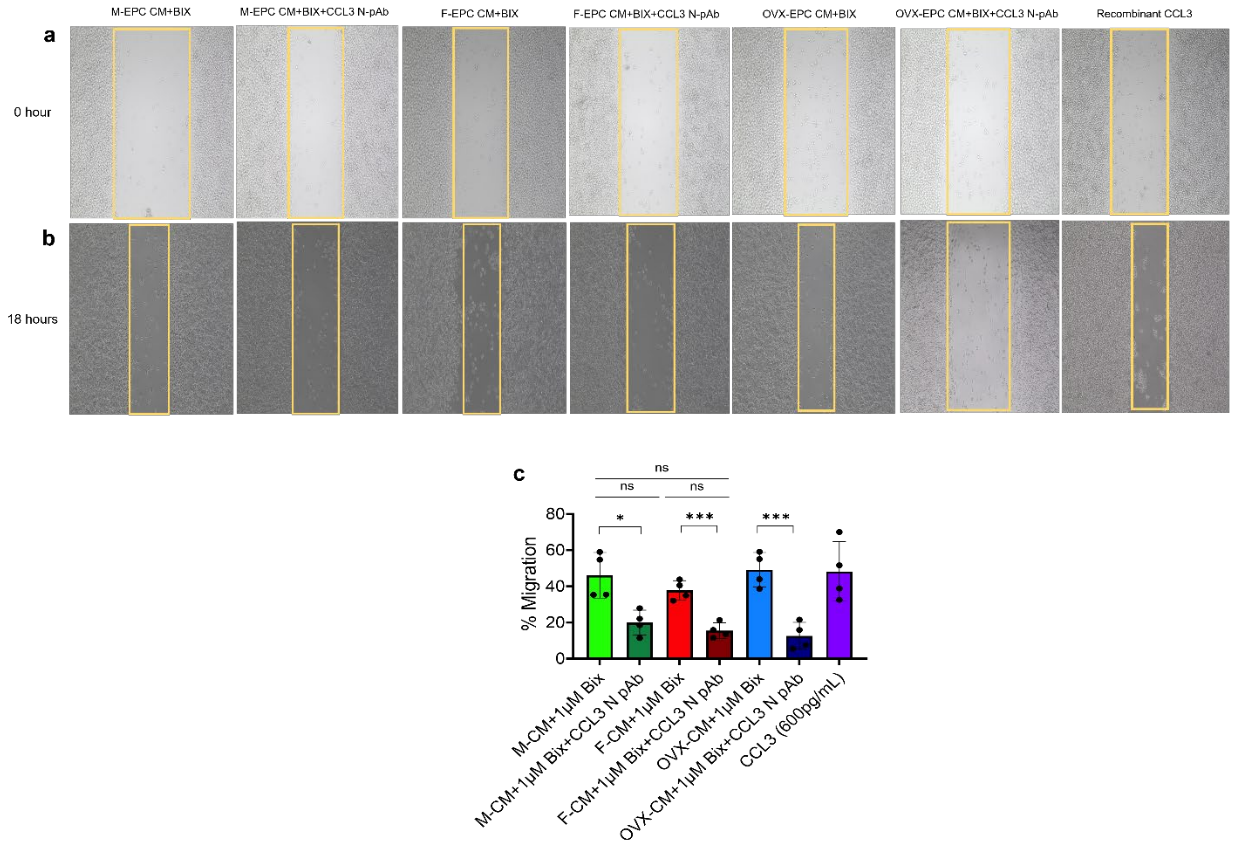

Supplementary Figure 9- Effect of BIX-01294 on sex dimorphic EPC secretome to promote monocyte migration. **a, b & c,** CMs from BIX-01294 treated differential EPCs promoted migration of Raw 264.7 cells in an *in vitro* scratch wound model. Neutralization of CCL3 in the BIX-treated CMs resulted in significant inhibition of monocyte migration at 18 hours. The addition of recombinant CCL3 (600 pg/mL) promoted the migration of monocytes equivalent to BIX-treated EPCs CMs. *P<0.01; **P<0.001; ***P<0.0005. 400× magnification. Data shown as mean ± s.e.m.

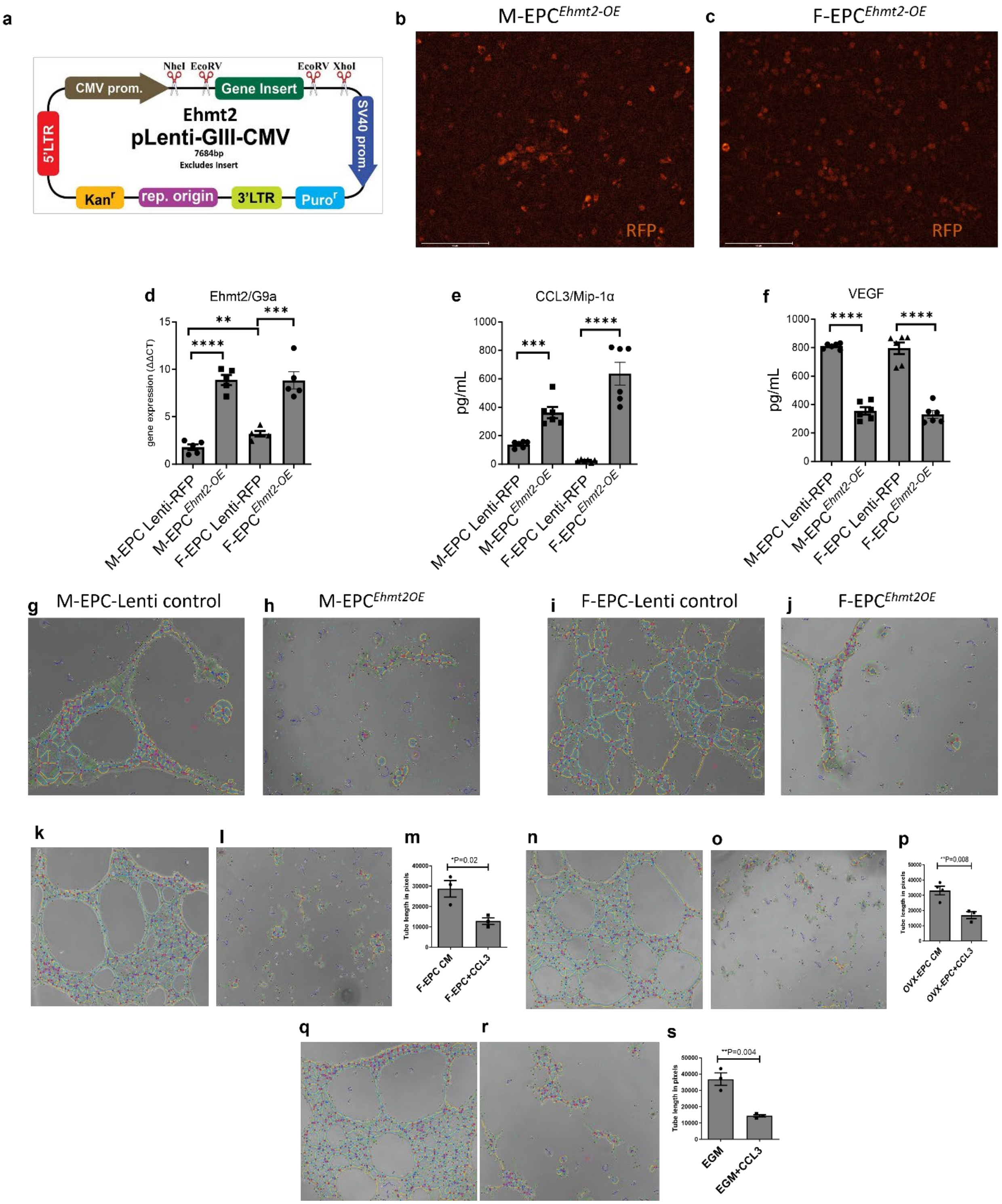

Supplementary Figures 10 – Effects of G9a overexpression in EPCs. a, Sequence map of CCL3-specific piLenti-overexpression vector. **b**, **c**, confirmation of transfection and

overexpression of G9a in M-EPCs and F-EPCs by RFP expression post 72 hours. Confirmation of G9a gene expression in M-EPCs and F-EPCs by RT-PCR (**d**). Secretion of CCL3 (**e**) and VEGF (**f**) in M-EPCs – Lenti control, M-EPC$^{Ehmt2\text{-}OE}$, F-EPCs - Lenti control and F-EPC$^{Ehmt2\text{-}OE}$. CM from M-EPC$^{Ehmt2\text{-}OE}$ (**h**) and F-EPC$^{Ehmt2\text{-}OE}$ (**j**) showed impaired tube formation compared with M-EPC – Lenti control (**g**) and F-EPC – Lenti control (**i**). 1000× magnification. Data shown as mean ± s.e.m.

CCL3 is a potent inhibitor of angiogenesis. F-EPC CM (**k, m**), OVX-EPC CM (**n, p**) and EGM (**q, s**) efficiently promoted tube formation of MCECs. Addition of CCL3 (100 pg/mL) in F-EPC CM (**l, m**), OVX-EPC CM (**o, p**), or EGM (**r, s**) significantly inhibited tube formation compared with CMs without CCL3. *P<0.01; **P<0.001. 1000× magnification. Data shown as mean ± s.e.m.

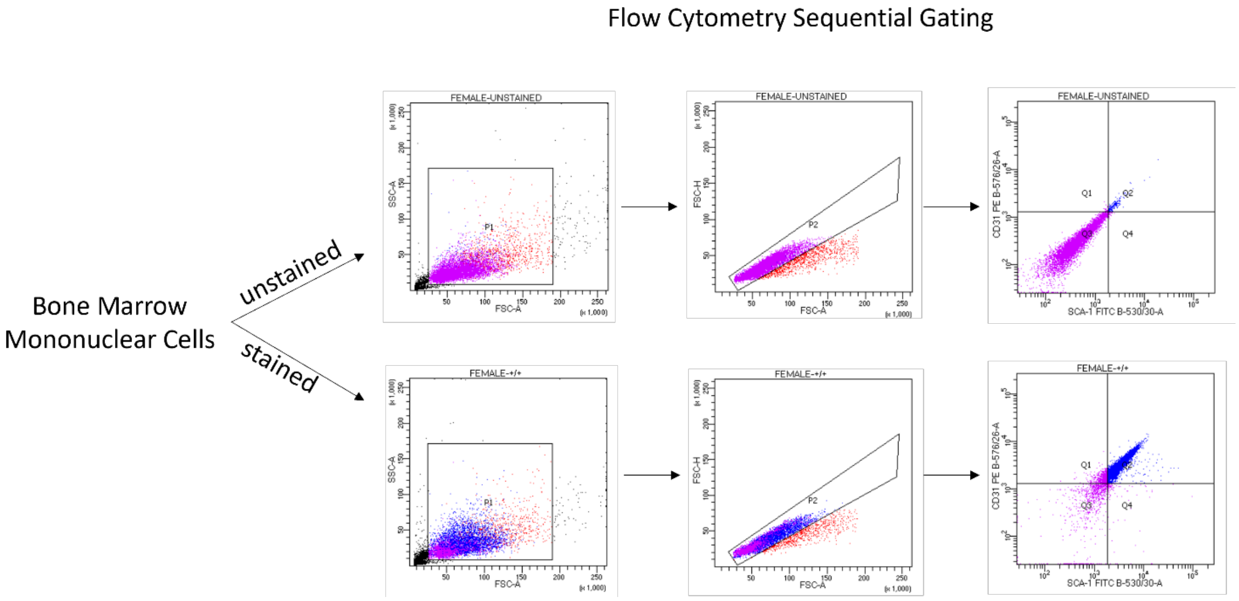

Supplementary Figure 11 – Graphical sequential gating strategy for flow cytometry analysis. Graphical abstract showing the sequential gating strategy for bone marrow

mononuclear cells (BMMNCs) isolated from male, female and OVX mice. Strategy shows gating for unstained BMMNC as well as for BMMNCs stained with CD31-PE and Sca-1-FITC antibodies.

Supplementary Table 1: qRT-PCR primer sequences (Mouse)

	List of primers (Mouse)		
Gene Symbol	Forward	Reverse	SOURCE
VEGF-A	AGGCTGCTGTAACGATGAAG	TCTCCTATGTGCTGGCTTTG	INTEGRATED DNA TECHNOLOGIES, INC.
VEGFR2	GAGCTCTCCGTGGATCTGAAA	AACAAAGCCTGAGCTGGCAG	INTEGRATED DNA TECHNOLOGIES, INC.
Angpt-1	AACCGAGCCTACTCACAGTACG	GCATCCTTCGTGCTGAAATCGG	INTEGRATED DNA TECHNOLOGIES, INC.
eNOS	TCTGCGGCGATGTCACTATG	CATGCCGCCCTCTGTTG	INTEGRATED DNA TECHNOLOGIES, INC.
IL1β	TGGACCTTCCAGGATGAGGACA	GTTCATCTCGGAGCCTGTAGTG	INTEGRATED DNA TECHNOLOGIES, INC.
IFNγ	GGAGGAACTGGCAAAAGGATG	GACCTGTGGGTTGTTGACCT	INTEGRATED DNA TECHNOLOGIES, INC.
TNFα	GGCTGCCCCGACTACGT	AGGTTGACTTTCTCCTGGTATGAGA	INTEGRATED DNA TECHNOLOGIES, INC.
CCL3	ACTGCCTGCTGCTTCTCCTACA	ATGACACCTGGCTGGGAGCAAA	INTEGRATED DNA TECHNOLOGIES, INC
CCL5	CCTGCTGCTTTGCCTACCTCTC	ACACACTTGGCGGTTCCTTCGA	INTEGRATED DNA TECHNOLOGIES, INC
CXCL9	CCTAGTGATAAGGAATGCACGATG	CTAGGCAGGTTTGATCTCCGTTC	INTEGRATED DNA TECHNOLOGIES, INC
Arg-1	AGGAGGACAGTCGCGTACA	AGCTCGGCCGCGACA	INTEGRATED DNA TECHNOLOGIES, INC
IL-10	TAAGGCTGGCCACACTTGAG	GTTTTCAGGGATGAAGCGGC	INTEGRATED DNA TECHNOLOGIES, INC
